# Have the environmental benefits of insect farming been overstated? A critical review

**DOI:** 10.1111/brv.70076

**Published:** 2025-10-28

**Authors:** Corentin Biteau, Tom Bry‐Chevalier, Dustin Crummett, Katrina Loewy, Ren Ryba, Michael St. Jules

**Affiliations:** ^1^ The Insect Institute 2424 E York St. Unit 204 Philadelphia PA 19125 USA; ^2^ AgroParisTech‐INRAE, BETA Université de Lorraine Nancy France; ^3^ Chaire Economie du Climat, Palais Brongniart 28 Pl. de la Bourse Paris 75002 France; ^4^ Animal Ask Unit 10, The Linen House, 253 Kilburn Lane London W10 4BQ UK

**Keywords:** black soldier fly, circular economy, cricket, insect farming, life cycle assessment, yellow mealworm

## Abstract

Insect farming is frequently promoted as a sustainable food solution, yet current evidence challenges many environmental benefits claimed by industry proponents. This review critically examines the scientific foundation for assessing the environmental impacts of insect farming in both human food and animal feed applications. Our analysis reveals substantial limitations in existing research. Most studies have been conducted in small‐scale settings, which may not accurately reflect real‐world, industrial conditions. There are significant uncertainties, with many authors highlighting the fact that the future environmental impact of large‐scale insect production is unknown. This is especially true given claims that insects can be fed on food waste and that insect frass can be used as fertiliser, both of which have considerable challenges to overcome at scale. Furthermore, insect‐based foods predominantly substitute for plant products with limited environmental impact rather than meat, while evidence indicates that insect feed and pet food applications, when not utilising genuine food waste, generate greater environmental impacts than conventional alternatives. By providing a comprehensive overview, this review highlights key areas for further research and ensures policymakers have a clearer picture of the remaining uncertainties surrounding this emerging industry.

## I. INTRODUCTION

The current food system contributes significantly to biodiversity loss, deforestation, and climate change (Poore & Nemecek, 2018; Xu *et al*., 2021). Despite occupying 77% of global agricultural land, livestock and feed crops contribute merely 18% of human caloric intake and 37% of dietary protein worldwide. Insect‐based products are frequently cited as a sustainable alternative to meat, feed, or pet food. While the industrialised farming of insects as food and feed is a new phenomenon, the practice of eating insects has a deep‐rooted history and is practised by over two billion people globally (van Huis & FAO, 2013).

In recent years, insect farming has witnessed substantial growth, attracting heightened interest from industrial, governmental, and academic sectors (Sogari *et al*., 2022). Over $1.5 billion has been invested in the insect farming industry (Watson, 2024), leading to the construction of large‐scale automated facilities capable of producing trillions of insects, with more such facilities in development. Legislative changes in regions like the European Union have created a more favourable environment for expanding the use of insects as human food, pet food and animal feed. Predominant species in insect farming include the yellow mealworm (*Tenebrio molitor* L.), black soldier fly (*Hermetia illucens* L.) larvae (BSFL), and the house cricket (*Acheta domesticus* L.).

Pet food currently leads the insect‐based product market, having captured approximately 50% of market share in 2020 (de Jong & Nikolik, 2021). While earlier forecasts suggested that aquaculture feed would become the dominant sector by 2030, recent industry movements challenge this outlook. Notably, market leader Ÿnsect has started pivoting away from the feed sector, redirecting its focus to the more profitable pet food segment (Reus, 2023). Insect‐based food products intended for human consumption only account for a very small proportion of investment: less than 5% in 2022 (Eurogroup for animals, 2023).

Insect‐based pet food primarily aims to replace conventional cat and dog food, generally focusing on the supposed health benefits or the perceived environmental advantages. Insect meal targets the replacement of conventional animal feed, especially in aquaculture, and to a lesser extent in poultry. It is mostly promoted as a substitute for fishmeal, which is associated with forage fish depletion, and soy meal, linked to deforestation (Oliva‐Teles, Enes & Peres, 2015). Although insect‐based foods are often presented as replacements for animal‐based products, 90% of them are not meat substitutes (IPIFF, 2020*a*). In addition, a core value of insect farming lies in its role as a potential waste‐management activity, and the fact that ‘waste’ from the insect‐processing stream can be used in non‐food products (Ojha, Bußler & Schlüter, 2020). In particular, insect frass could be used to replace conventional fertilisers, which are associated with significant environmental impacts.

In this article, we review the literature and critically examine the evidence that has been used to inform policy debates on the environmental impacts of insect agriculture. This report aims to provide an overview of the environmental impacts of the current large‐scale insect farming industry. Therefore, we focus primarily on data from Western countries, especially the EU and to a lesser extent the UK, where most large‐scale companies are located. While insect farming is present in other parts of the world, it is typically done on a smaller scale, with very different production contexts, although there is potential for large‐scale companies to expand into these regions in the future (Baiano, 2020).

Several arguments seem to support the sustainability of insect farming. Insects are believed to convert feed into protein more efficiently than conventional livestock, with a lower feed conversion ratio (van Huis & FAO, 2013; Halloran *et al*., 2016). Unlike mammals and birds, insects are exothermic, meaning they do not expend energy to regulate body temperature. Moreover, insects can potentially consume a variety of feed sources, including organic waste (Halloran *et al*., 2016). However, these characteristics do not inherently ensure the environmental friendliness of insect‐based products (Liverød, 2019; Lange & Nakamura, 2023).

Notably, recent research indicates that the industry makes very little use of food waste to feed insects due to several barriers, including nutritional and logistical challenges (Biteau *et al*., 2024*b*). Instead, it relies primarily on more expensive, higher‐quality ingredients such as commercial feed or agricultural co‐products that are suitable for direct consumption by other animals or, in some cases, humans. In this case, where insects eat feed‐grade products and are then themselves used as feed, insect farming may increase the environmental footprint of our food system by introducing an additional step in the food production chain. Therefore, this study focuses on ingredients that are currently representative of industry practices, while potential future improvements, such as using waste, are discussed separately.

Despite the potential of insect agriculture, significant gaps remain in the literature. Several studies highlight substantial uncertainties, noting that the future environmental impact of large‐scale insect production is largely unknown (Berggren, Jansson & Low, 2019; Lange & Nakamura, 2023). Although the European Commission has recently approved new uses for insect products, its experts acknowledge an ‘overwhelming lack of knowledge concerning almost every aspect of production’ (EU Platform on Sustainable Finance, 2021, p. 22).

This review examines the key drivers of the environmental impact of insect farming, followed by a critical evaluation of the empirical evidence regarding its environmental benefits. It identifies key factors and knowledge gaps in insect farming, and explores the environmental impacts of insect‐based products used as human food, livestock and aquaculture feed, pet food, and fertiliser. We also address potential biodiversity risks and pathogen concerns. Following this, we discuss potential ways of improving the sustainability of insect farming, focusing on waste utilisation and technological advancements, before briefly examining the economic outlook. Through a comprehensive review of current scientific literature, this study aims to identify critical knowledge gaps for future research and provide policymakers with a nuanced understanding of outstanding questions in the field.

## II. METHODS

The studies considered in this literature review were primarily retrieved from searches from *Google Scholar*, *Scopus*, *OpenAlex* and *ScienceDirect*, between August 2023 and September 2024. Other tools such as *SciSpace* and *Consensus* were also used. Combinations of the following key words were used: ‘insect farming’ (or ‘insect farm[s]’), ‘environmental impact[s]’, ‘Life Cycle Assessment’ (or ‘LCA’), ‘CO2’ (or ‘global warming’, or ‘greenhouse gas’). Terms like ‘black soldier fly’, ‘mealworm’, or ‘cricket’ were also used. All consulted references were in English or in French.

Additional studies were sourced by consulting the reference lists of reviewed papers or from our prior knowledge. Some studies were identified by searching the published work of notable authors in the field, such as Arnold van Huis, Sergiy Smetana, Dennis Oonincx, and Imke de Boer. Finally, colleagues working on alternative proteins also provided some resources. The references were prioritised according to publication year, with a focus on those published from 2020 onwards due to the rapidly evolving nature of insect farming. An effort was made to screen the most regularly referenced pre‐2020 studies.

**Fig. 1:**
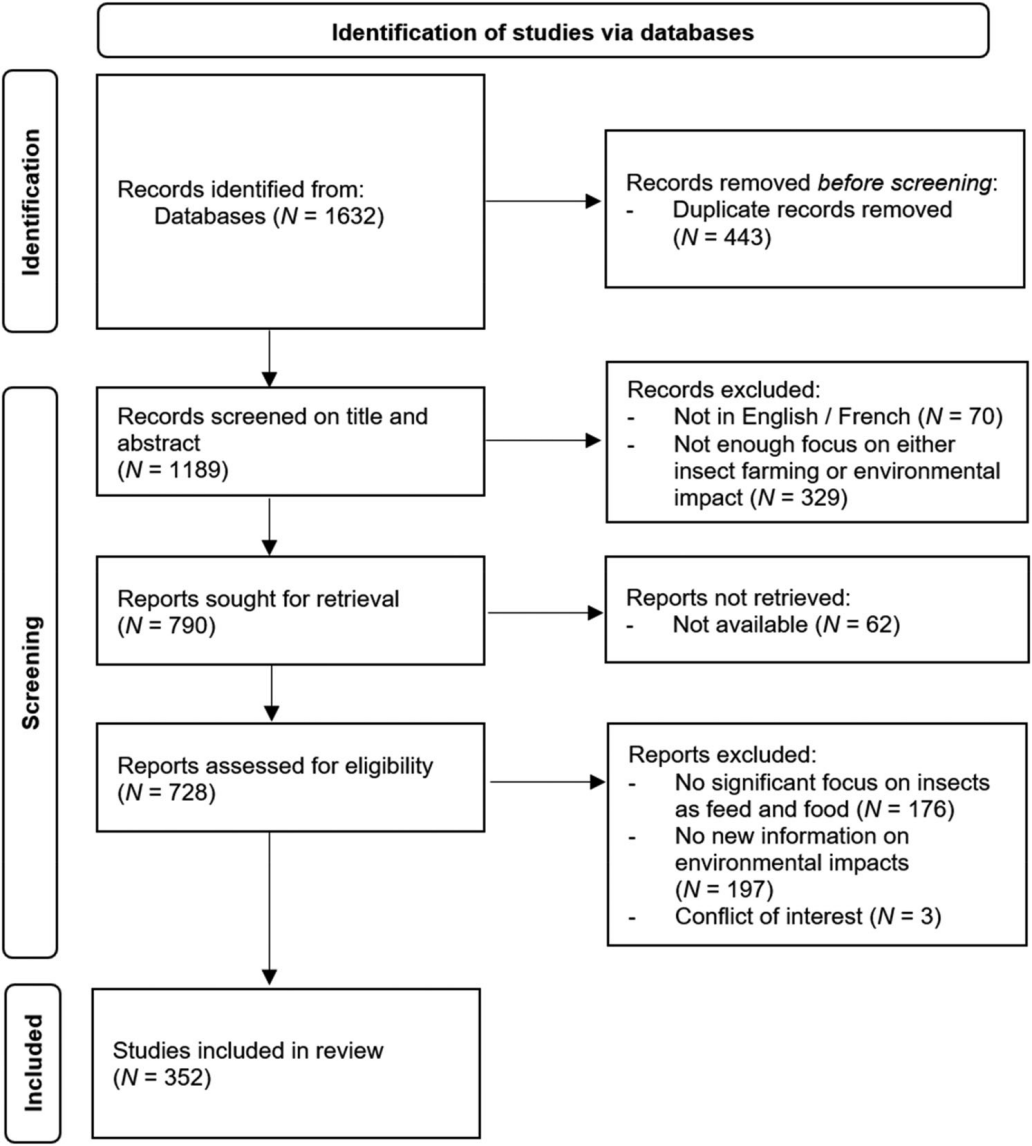
PRISMA 2020 flow diagram (Page *et al*., 2021) documenting study selection for inclusion in this review.

A PRISMA flowchart of the screening process is shown in Fig. 1. We focused on the use of insects as food and feed, as well as fertiliser. While peer‐reviewed journal articles were prioritised, reports from broader sources, such as non‐governmental organisations, were also considered due to the limited availability of certain data, especially on market funding. To avoid conflicts of interest, efforts were made to avoid relying on studies funded by companies from the insect industry.

The combined research found a total of 1189 studies. Following the removal of duplicates, an initial screening was performed on the title and abstract. Following this step, 329 papers were excluded due to not having a significant focus on insect farming or its environmental impact, 62 papers were excluded as the full text could not be retrieved, and 70 papers were excluded as they were not in English or French.

A more advanced screening was performed on the remaining papers, in which the content of the paper was examined to assess its relevance to the literature review. At this stage, 176 papers were excluded as their focus was not on insects as feed, food or fertiliser. While they did mention insect farming, it was often a brief mention as a potential solution, without delving significantly into its environmental impact. This was the case for many papers on waste management or aquaculture feed. A further 197 papers were excluded due to not having a focus on environmental impacts. Their focus was rather on nutritional, economic, or social aspects of insect farming. Sometimes, they provided claims about the sustainability of the sector, but did not add new data such as greenhouse gas (GHG) emissions obtained in farms. Finally, three papers were excluded due to a potential conflict of interest.

The inclusion criteria were: (*i*) studies that performed a LCA with detailed numerical data on the environmental impact of insect farming; (*ii*) studies that provided a review of other LCAs; (*iii*) studies that provided detail and new information on the environmental impacts of the industry, whether quantitative or not; (*iv*) reviews comparing the environmental impacts of several products to which insects contribute, e.g. meat substitutes or aquafeed; (*v*) studies focusing on the broader context with a relevant perspective on the sustainability of insect farming, including studies providing information on social, economic or logistical constraints. After study screening, a total of 352 papers were included in the review.

## III. GENERAL CONSIDERATIONS REGARDING THE ENVIRONMENTAL IMPACT OF INSECT FARMING

The primary determinants of environmental impact in insect farming are the production of the substrate (the feed given to insects) and the energy required for rearing and processing insects (Smetana, Schmitt & Mathys, 2019; Vauterin *et al*., 2021).

### (1) Substrate

The feed provided to insects is the most significant contributor to the environmental impact of insect‐based products (Oonincx & de Boer, 2012; Lundy & Parrella, 2015; Halloran *et al*., 2016; Salomone *et al*., 2017; Oonincx, 2021; Vauterin *et al*., 2021; Sogari *et al*., 2023*b*). By‐products like organic waste usually yield better environmental outcomes than conventional substrates like grains (Halloran *et al*., 2016; Smetana *et al*., 2023*a*; Paris *et al*., 2024). However, this is not always the case (Shockley & Dossey, 2014; Beyers *et al*., 2023).

Several factors are involved in the environmental impact of the substrate, including its nutritional content, cost, environmental footprint, the growth rate of the insects, and whether it constitutes an unused side stream of another process (Sogari *et al*., 2023*b*). Generally, high‐quality substrates such as grains lead to faster growth in insects, but their production often entails a higher environmental impact and may compete with their use as human food or animal feed (Smetana *et al*., 2016; Spykman *et al*., 2021). On the other hand, lower‐quality substrates like manure, household waste or potato peelings typically have a lower environmental footprint but can lead to smaller insects or extended growth periods, which might increase resource consumption during the growth phase and negate any environmental benefits (Smetana *et al*., 2016; Smetana, Spykman & Heinz, 2021*b*; Bosch *et al*., 2019; Spykman *et al*., 2021; Beyers *et al*., 2023). For example, the larval period of the yellow mealworm ranged from 44 days on a grain‐based diet (wheat bran mixed with distillers dried grain) to 227 days on a low‐protein diet (Bordiean, Krzyżaniak & Stolarski, 2022). Due to these extended rearing times, Ites *et al*. (2020) failed to identify economically viable ways to rear mealworms on low‐value waste. Crickets, for similar reasons, are rarely fed on waste (Lundy & Parrella, 2015; Skrivervik, 2020). Some species, however, such as BSFL, are better suited to a broader range of low‐value organic waste, including rice straw or catering waste. More generally, the variability of organic waste complicates identification of an optimal feed composition, and longer growth cycles compromise economic feasibility (Shurson, 2020; Van Peer *et al*., 2021). There is often a trade‐off between economic and environmental performance. The environmental impacts of using waste, with the potential benefits of waste removal, are detailed in Section IX.1.

There are other drawbacks to the use of waste as a substrate. Insects may experience increased mortality rates when fed with unprocessed waste, as has been observed in commonly farmed species, including crickets reared on municipal waste (Lundy & Parrella, 2015) and BSFL reared on manure (Miranda, Cammack & Tomberlin, 2020). The yellow mealworm has limited suitability for rearing on organic waste and manure substrates (Le Féon *et al*., 2019; Harsányi *et al*., 2020). Due to health and safety concerns, regulatory constraints in the EU, USA and UK limit the use of mixed household waste as substrate, despite it representing 70% of food waste in the EU (Salemdeeb *et al*., 2017; Mancini *et al*., 2022). Regulatory changes in favour of a circular economy may occur in the future, although predicting these changes is challenging. Furthermore, since the nutritional profile of insect meal depends on components of the insects' diet, waste‐fed insects may be unable to deliver the stable, consistent nutritional content required by the aquaculture and livestock industries (Sogari *et al*., 2023*b*).

As a result, most insect farming companies do not use organic waste but instead rely on high‐quality, often grain‐based, substrates (IPIFF, 2018; Gibson, 2022; Faes, 2022; Biteau *et al*., 2024*b*). These substrates are already widely used as animal feed (Heidari, Gandasasmita & Pelletier, 2021), meaning that their use in insect agriculture competes with these established sectors.

### (2) Energy use

Energy use in insect farming can vary significantly with factors such as building design, location, substrate type, and processing technology. For example, the impact of energy use on GHG emissions is higher in carbon‐intensive electricity grids (Kleyn, 2023). Some studies indicate that heating is the primary driver of energy consumption (van Zanten *et al*., 2015; Salomone *et al*., 2017; Smetana *et al*., 2019), while others suggest that processing accounts for over half of energy use (Thévenot *et al*., 2018), or find no single dominant factor (Kleyn, 2023). Conversely, a recent study argued that processing the substrate serving as insect feed accounts for the highest non‐renewable energy use and freshwater withdrawal, and that feed processing contributes to only 1–5% of the total environmental impact (Smetana, Ristic & Heinz, 2023*b*).

As insects are cold‐blooded, they require an external source of heat to regulate their temperature, with optimal rearing conditions typically being 25–30 °C and 50–70% humidity, varying by species (Odhiambo, Ochia & Okuto, 2022; Rho & Lee, 2023; Korir *et al*., 2024). Growth rates are temperature dependent; for example, crickets grow in 8 weeks at 30 °C but take 8 months at 18 °C (Ayieko *et al*., 2015). Longer growth periods increase energy, feed, and water requirements, raising environmental impacts (Halloran *et al*., 2016).

The geographical location of a factory significantly affects the energy needed for temperature control (Halloran *et al*., 2016; Maiolo *et al*., 2021). Insects can be reared outdoors in tropical climates such as Thailand (Halloran *et al*., 2017), but heated facilities are necessary in the cooler climates of the EU or UK, increasing energy use (Liverød, 2019). Maintaining optimal temperatures year‐round, especially during winter, requires substantial energy and contributes to GHG emissions, although using renewable energy sources or residual heat from nearby facilities can help mitigate this impact (Quang Tran, Van Doan & Stejskal, 2022). Heating costs accounted for 19% of GHG emissions of insect production systems in the UK (Suckling *et al*., 2020) and up to 65% for those in Austria (Dreyer *et al*., 2021). Given that temperature and energy sources vary by location, findings from one context may not be directly translatable to another.

The costs of processing, including drying of insect products, are often highlighted in terms of energy consumption (Salomone *et al*., 2017; Roffeis *et al*., 2017,2020; Mertenat, Diener & Zurbrügg, 2019; Bava *et al*., 2019; Ites *et al*., 2020). Smetana *et al*. (2021*b*, p. 563) described these as having “relatively high energy demand and could result in high associated environmental impacts”. Processing, including drying, can account for 7–45% of electricity use (Bava *et al*., 2019), more than half of energy consumption (Thévenot *et al*., 2018) and up to 20% of the overall environmental impact (Goyal *et al*., 2021).

Other aspects of energy use make a smaller contribution to the environmental impact of insect production. The impact of transportation and the insect reproduction process is comparatively minor (Smetana *et al*., 2021*b*). The environmental cost of constructing the facility is often not assessed (Spykman *et al*., 2021), but has been presumed to be marginal based on older studies, although this may not be representative of emerging fully automated industrial processes (Halloran *et al*., 2016).

### (3) Major knowledge gaps

An evaluation of the environmental impact of insect farming must acknowledge the limitations and gaps in existing literature.

For crickets, one of the species most commonly reared in insect farms, we found almost no reliable assessments of environmental impacts and no estimate for pet food products in industrialised Western countries. The few existing studies are difficult to compare because of wide variation among them (e.g. see Table 1). A LCA by Halloran *et al*. (2017) for a medium‐sized farm in Thailand indicated lower GHG emissions for crickets compared to meat, and this finding has been extensively cited. However their data were from crickets in a tropical outdoor setting that were fed on grain supplemented with pumpkins; these conditions differ significantly from potential farming conditions in Western countries, where indoor heating is necessary. Suckling *et al*. (2020) carried out the first commercial‐scale insect LCA in the UK, and found GHG emissions nearly ten times higher than reported by Halloran *et al*. (2017).

**Table 1:** Comparison of two life cycle assessments performed on crickets and their relevance for determining the environmental impact of crickets in industrialised production in Western countries.

Study	Halloran *et al*. (2017)	Suckling *et al*. (2020)
Location	Thailand	UK
Insect species	*Acheta domesticus* and *Gryllus bimaculatus*	*G. bimaculatus*
Market	Human consumption	Live pet food
Greenhouse gas emissions	4.2 kg CO2e per kg of protein	33.49 kg CO2e per kg of protein
Study strengths	Includes the most commonly reared cricket species (*A. domesticus*)	Representative of business conditions in the UK Heating requirements representative of Europe
Study limitations	High ambient temperatures with no energy required for heating Medium‐scale farm Farms in Thailand have very diverse farming systems (more than 20,000 farms), and the one studied may not be representative Does not represent business conditions in Europe (outdoor setting, factories are less automated partly because labour is cheaper)	Small‐scale farm Several inefficiencies due to the need to sell crickets alive, which complicates storing Inclusion of the carbon emissions from frass, with several uncertainties

This example illustrates the current gap in understanding the environmental impacts of insect farming. Compared to well‐established agricultural sectors, data availability for insect agriculture is sparse (Bosch *et al*., 2019; Ites *et al*., 2020; Smetana *et al*., 2021*a*), and producers often do not make their data public. As of 2025, less than a dozen insect‐related LCAs have been conducted on actual farms in Western countries, including Suckling *et al*. (2020). Most LCAs have focused on cradle‐to‐gate analyses, often excluding factors like distribution and transportation costs (van Huis *et al*., 2021).

Our current understanding of the environmental impacts of insect agriculture thus inherently relies on a small set of studies. Older studies (e.g. van Huis & FAO, 2013; Smetana *et al*., 2016), are widely cited, but are increasingly out of date in this rapidly evolving field and their assumptions may be out of line with current business practices. The LCA cited most often was carried out by Oonincx & de Boer (2012), but was based on a production system that is not representative of actual large‐scale operations, as they considered insects fed with fresh carrots and mixed grains to produce live or frozen insects as food for birds or reptiles. Several studies were conducted in pilot or small‐scale facilities, processing only 0.02–1 tonne of dried insect biomass daily, adding further uncertainty regarding their applicability to larger‐scale commercial production (Smetana *et al*., 2019; Van Peer *et al*., 2022). Shine (2020) emphasised that, due to the complexity and diversity of factors involved, LCAs should be conducted for each product under locally relevant conditions. Without such tailored assessments, current numbers should be viewed ‘more as enthusiastic speculation than actual demonstrable figures’ (Shine, 2020, p. 71).

Several companies, such as Protix and Ÿnsect, provide case studies on their production methods, often highlighting highly favourable environmental outcomes. However, these studies are not subject to critical peer review, and the lack of access to source data, along with figures that diverge significantly from the scientific literature (e.g. Protix claim that its PureeX insect meat uses 99.8% less water than poultry meat), raises concerns about their reliability. During preparation of this review, we contacted several major insect farming companies. Four of these did not respond, and the one that did (Protix) declined to share their environmental data with us for reasons of confidentiality. Future research would benefit from increased collaboration between industry and researchers, which would provide more reliable metrics and enhance transparency.

## IV. ENVIRONMENTAL IMPACT OF INSECT‐BASED FOODS

The insect food market currently attracts only a minimal share of funding in the insect sector. According to a Rabobank report, ‘their market share is negligible, and opportunities, at least for now, are limited’ (de Jong & Nikolik, 2021, p. 2). Nevertheless, edible insects represent the most publicly visible segment of this sector, including in the mainstream media, shaping how the public thinks about insect farming.

For insect‐based foods to be considered more sustainable than existing foods, they must have a lower environmental footprint than the foods they aim to replace. Therefore, assessing the ecological impact of insects as food requires understanding the products that they are substituting. Most scientific research on this topic compares insects to conventional meat (Bordiean *et al*., 2020; Capestany, 2021; Smith *et al*., 2021; Abdullahi, Igwe & Dandago, 2022; Vinci *et al*., 2022; Vale‐Hagan *et al*., 2023) and, to a lesser extent, meat alternatives (Hadi & Brightwell, 2021; Smetana *et al*., 2021*a*). Positive comparisons of environmental impacts of insect‐based foods compared to meat are regularly presented by insect‐farming companies. However, while insects offer an additional source of protein, their adoption may not always result in reduced meat consumption (Halloran *et al*., 2016; Shine, 2020; Cottrell *et al*., 2021). Due to issues with consumer acceptance, many edible insects in Western countries are predominantly used in items such as snacks, which do not serve the same nutritional role as meat. This presents an important consideration: if insects do not replace meat, what is their contribution to more sustainable food systems?

### (1) What are insect‐based foods competing with?

**Fig. 2:**
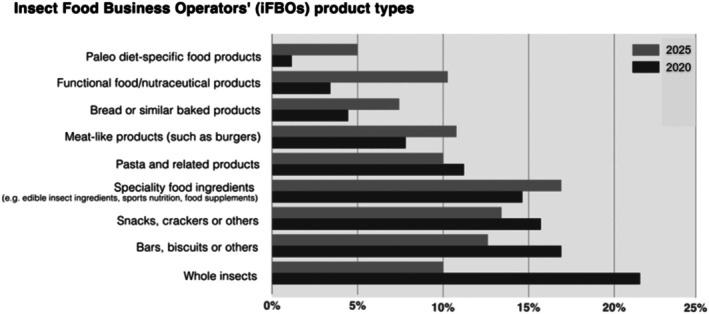
Market share of different insect‐as‐food product types, estimated in 2020 (light bars) and projected for 2025 (dark bars). Data from IPIFF (2020a).

The most commonly found insect‐based products in Europe and North America are whole insects, energy bars, biscuits and cookies, snacks such as crisps or crackers, protein powder, pasta, burger patties, or bread (Skrivervik, 2020; Mancini *et al*., 2022; Żuk‐Gołaszewska *et al*., 2022; Sogari *et al*., 2023*a*). The International Platform of Insects for Food and Feed (IPIFF, 2020*a*) estimated that in 2025, whole insects would constitute close to a quarter of the market, followed by bars, snacks, speciality food ingredients (e.g. food supplements) and pasta (Fig. 2).

Thus, except for burger patties or sausages, insect‐based foods do not fill the same culinary role as meat. Meat‐like products only accounted for 8% of the insect food market in 2020, a figure expected to remain below 12% in 2025 (IPIFF, 2020*a*). Instead, insects mostly replace plant‐based products, like maize in tortilla chips or chickpea flour in protein‐supplemented pasta or bread. These ingredients usually have a much lower environmental footprint than meat (Poore & Nemecek, 2018; Ritchie, Rosado & Roser, 2022), therefore incorporating insects could increase rather than decrease the environmental impact of such foods (Shine, 2020). Even when insect‐based products substitute for animal‐sourced foods, they may still face competition from more sustainable plant proteins (Lucas, Guo & Guillén‐Gosálbez, 2023). For example, while insect protein powder could replace whey protein powder, plant‐based protein supplements might offer a more environmentally friendly alternative. In fact, plant‐based options accounted for nearly 40% of the protein supplement market in 2023, with projections indicating further growth (Grand View Research, 2023).

Therefore, comparisons of the sustainability of insect‐based foods with meat, as is commonly done in the literature (Baiano, 2020; Kemsawasd *et al*., 2022; Illa & Yuguero, 2022; Ros‐Baró *et al*., 2022), are not an appropriate perspective. Similarly, most companies selling insect snack bars promote them as environmentally friendly by comparing them to animal products (Andreani, Sogari & Banović, 2024), although there is no evidence showing they are more sustainable than traditional snack bars (e.g. muesli bars). There is a risk of fostering a perception that insects are inherently sustainable, even when used in desserts and snacks, rather than specifically as meat replacements. A solution to avoid misleading ideas could be a standardised carbon label on the packaging of food products, which could help consumers pick foods with the lowest environmental footprint (Taufique *et al*., 2022).

On the other hand, some authors argue that these products may serve as a gateway, fostering acceptance for a broader range of insect‐based foods. Introducing novel foods like insects in familiar contexts could potentially help create more positive expectations in the future (van Huis & Rumpold, 2023). However, this gateway hypothesis has not yet received empirical support (House, 2019). Another suggestion is that additional protein in the diet from insects added to food like pasta, cookies, or protein bars, could lead to reduced meat consumption elsewhere in the diet. However, we have not encountered any evidence to support this claim. One study found that fortified food consumption in Finland did not significantly alter nutrient intake, including meat consumption (although the study was not limited to protein supplements) (Hirvonen *et al*., 2012).

### (2) Consumer acceptance of different products

The industry's focus on incorporating insects into familiar processed products aims at increasing consumer acceptance (Mancini *et al*., 2022; Żuk‐Gołaszewska *et al*., 2022). Studies indicate that Western consumers are less likely to consume unprocessed insects where parts like the head or legs are visible (Schösler, de Boer & Boersema, 2012; Ruby, Rozin & Chan, 2015; Hartmann & Siegrist, 2017).

Most research assesses the overall acceptability of insect‐based foods, often without making specific distinctions. Among studies that focused on specific products, the most commonly analysed products are burgers, bars, crisps, biscuits, and bread, with only burgers representing a direct meat substitute (Mina, Peira & Bonadonna, 2023). For example, Lombardi *et al*. (2019) explored consumer willingness to pay for insect‐based products, highlighting their environmental benefits compared to pork. However, the products in their study were cookies, pasta, and chocolate bars, not sausages. One survey found that “consumers were most willing to accept insects in snacks (37%), main dishes (26%) and desserts (23%), and they were least inclined to accept insect‐based salads (7%), soups (6%) and unprocessed insects (1%)” (Caparros Megido *et al*., 2024, cited by Żuk‐Gołaszewska *et al*., 2022). This implies that even if a study reports a moderate or high acceptance rate for insect consumption, it may not be representative of all product types.

It should also be noted that a willingness to pay is not a willingness to substitute, especially if insect‐based products remain relatively expensive. The success of insects as a meat replacement implies the disadoption of meat, an unspoken assumption poorly addressed in the literature (Cottrell *et al*., 2021). In an experiment led by Michel & Begho (2023) in the UK, 248 consumers were presented with a choice between different types of sausages: pork‐based, cricket‐based, and hybrid varieties, each with a specified price. The findings revealed that insect‐based sausages faced significant price penalties compared to pork‐based products, meaning that most participants showed a lower willingness to pay for these products, possibly preferring them only when priced lower than pork‐based options. The price penalty, while variable, was significant across all consumer groups, including environmentally conscious individuals with low food neophobia, even after they were informed about health and environmental benefits.

Young people are ‘most willing to consume insects if incorporated into energy bars, cereals, and sweet bakery products’ according to Palmieri *et al*. (2023) (cited by Michel & Begho, 2023, p. 8). However, this also means that meat‐like insect products are not among the ones these consumers are most ready to try, especially as they have limited similarities with meat from sensory, functional, usability and symbolic points of view (Shine, 2020). Replicating the taste, smell, and flavour of animal‐based products remains a significant challenge (Malila *et al*., 2024). Insects are also not expected to reach price parity with meat before plant‐based proteins or single‐cell proteins (Malila *et al*., 2024). More generally, the idea of a gateway dish, leading to broader acceptance, lacks empirical support. Past examples of new ingredients gaining in popularity, such as raw fish in sushi, tend to show that instead, many elements are required, such as skilled chefs, new recipes or cultural contexts in which to try the new ingredient (House, 2019). Consequently, it seems unlikely that insect products will be popularised through a gateway dish, such as snacks or desserts. Note that recent efforts have been made to provide new recipes, with some restaurants trying to improve cultural acceptance.

### (3) Overview of life cycle assessments of insects as food

Table 2 provides an overview of the environmental impacts of insect production, summarising life cycle assessments (LCAs) that focus on insects as food. The results show substantial variability, and in some cases, only a single study was available for a given metric.

**Table 2:** Environmental impacts of insects as food. Comparison between different life cycle assessments (LCAs), highlighting the context and limitations of each study, with data from commercial and pilot‐scale settings (excluding laboratory contexts).

Study and location	Species	Greenhouse gas emissions (kg CO2e per kg)	Land use (m^2^ per kg)	Water depletion (m^3^ per kg)	Energy use (MJ per kg)	Context	Study limitations
**kg of wet matter**							
Oonincx & de Boer (2012) – Netherlands	*Tenebrio molitor*	2.7^a^	3.6	NA	34.0	Small scale: 83 tons of fresh insects annually	Small scale, use of fresh carrots in the substrate, slower development cycles than reported elsewhere
Halloran *et al*. (2017) – Thailand	*Acheta domesticus* *Gryllus bimaculatus*	2.3–2.6	NA	0.42	NA	Pilot: 36.7 tons of insects annually as human food	Outdoor setting in warm country, little automation due to cheap labour
**kg of protein**							
Halloran *et al*. (2017) – Thailand	*A. domesticus* *G. bimaculatus*	4.2	NA	0.71	NA	Pilot: 36.7 tons of insects annually	Outdoor setting in warm country, little automation due to cheap labour
Mungkung & Phetcharaburanin (2023) – Thailand	*A. domesticus*	4.6 (frozen) –11.3 (powder)	NA	NA	NA	Average of 36 small‐, medium‐, and large‐sized farms	Outdoor setting in warm country, little automation due to cheap labour
Dreyer *et al*. (2021) – Austria	*T. molitor*	20.4	22.38	NA	213.7	Small‐scale production	Small scale, use of organic feedstuff
Nikkhah *et al*. (2021) – South Korea	*Protaetia brevitarsis seulensis*	15.93	−0.20^b^	NA	64.6	Small‐scale unit: 1 ton per year	Very small scale, unusual species
Paris *et al*. (2024) – Canada	*T. molitor*	14.94	NA	NA	NA	Grain‐based feed scenario, small‐scale producers	Small scale, chicken feed as a substrate (using food waste has a lower impact), clean energy grid
Vauterin *et al*. (2021)	*Various*	12.4–25.8	NA	NA	NA	Mean values from the two scenarios. Uses data from other studies	Based on other studies that focused on both food and feed

NA denotes that a study did not include this variable in its LCA.

^a^ Modahl & Brekke (2022) found 20% greater land use and CO2e per kg dry protein using data reported in these studies when using a lower nitrogen‐to‐protein conversion factor of 4.76 that is more appropriate for insects.

^b^ Nikkhah *et al*. (2021) explain that the negative land‐use value of −0.20 m^2^ reflects a savings compared with the alternative use of the substrate, in this case mushroom production waste and banana peels, which would otherwise have a negative environmental impact.

We considered including Smetana *et al*. (2021*a*), which presents an LCA of meat‐substitute burgers. The bug burger in their study had a substantially lower environmental impact than beef (approximately 1.1 kg CO2e per kg of patty). However, insects made up less than 10% of the burger patty, making it inappropriate for inclusion in this table.

Most studies report impacts per kilogram of protein. Emissions for insects as food range from 4.2 to 25.8 kg CO_2e_ per kg protein (Table 2). Land use requirements vary widely, with one study estimating 22.38 m^2^ per kg protein, while another study in South Korea even reported slightly negative values (−0.20 m^2^/kg protein), reflecting avoided impacts by using mushroom and banana peel waste instead of conventional waste treatment. Energy use ranges from 64.6 to 213.7 MJ per kg protein, depending on production methods. Water footprint data are limited; Halloran *et al*. (2017) reported 0.71 m^3^ per kg protein in Thailand.

Per kilogram of wet matter, only two studies, one from the Netherlands (Oonincx & de Boer, 2012) and one from Thailand (Halloran *et al*., 2017), report a climate impact of 2.3–2.7 kg CO2e. For the other environmental metrics, with only a single estimate available, land use is 3.6 m^2^, water use is 0.42 m^3^, and energy use is 34.0 MJ per kg of wet matter.

No study to date has examined the environmental impacts of the most common insect‐based foods, such as bars or snacks, which prevents their inclusion as comparison points in the table. Nonetheless, a conservative comparison based on the Agribalyse database (Ademe, 2025) suggests that replacing plant‐based ingredients with insects would likely increase environmental impacts. For example, even with 2.3 kg CO2e per kg wet matter, which is at the lower end of the range observed, insects emit more GHGs than the food they likely replace: crackers (1.54 kg CO2e per kg wet weight), dry pasta (1.98 kg CO2e), flour (0.72 kg CO2e in cookies), or bread (0.78 kg CO2e). Given the limitations of this comparison, more comprehensive analyses are needed. These studies should conduct a comparison with protein‐fortified plant‐based products if relevant, and account for the specific ingredients that insects replace in each product (e.g. in chocolate bars, insects do not replace the chocolate content).

Many studies and companies compare insect farming with conventional livestock, although only a small fraction of insect‐based foods serve as meat substitutes. When insects do replace livestock, they often show environmental benefits (Oonincx, 2021; Vauterin *et al*., 2021; Vinci *et al*., 2022; Hunter, 2024). For example, compared with values for livestock from Smetana *et al*. (2023*a*), based on the Agrifootprint database, Table 2 shows that insects emit less CO2e per kg wet matter than beef (35.0), pork (6.95), and poultry (5.97). They also require less land than beef (23.1 m^2^), pork (6.28 m^2^), and poultry (4.64 m^2^). Energy use is lower than that of beef (104.0 MJ/kg), but higher than that of pork (28.3 MJ/kg) and poultry (23.8 MJ/kg). Water use was higher than that of beef (0.25 m^3^/kg), pork (0.05 m^3^/kg), and poultry (0.067 m^3^/kg) in the only available study assessing this criterion. However, the environmental performance of insect farming is highly context dependent. In some cases, insect‐based foods exhibit a higher impact than chicken or pork, particularly in colder or carbon‐intensive countries (Vauterin *et al*., 2021; Sillman, 2021). For example, mealworms reared on grain‐based feed in Canada exhibited emissions similar to poultry, which emits 15.1 kg CO2e per kg protein (Paris *et al*., 2024).

### (4) Comparison with alternative proteins

Efforts to reduce the substantial environmental impact of our food system have led to the exploration of various alternatives to meat, trying to replicate its sensory and nutritional properties while minimising environmental impacts. This group includes plant‐based meat substitutes, cultivated meat or single‐cell proteins. In a recent review assessing the environmental impacts of different meat substitutes, Smetana *et al*. (2023*c*) concluded that, when evaluated on a per kg of protein basis, insects generally exhibit a low environmental impact, but are outperformed by plant‐based substitutes (although note that few studies are available). In another assessment that included environmental impact, consumer acceptance, animal welfare and scalability, Bry‐Chevalier (2025) concluded that insect farming demonstrates limited environmental promise compared to the other alternative proteins considered (plant‐based meat substitutes, cultivated meat, single‐cell proteins).

Direct comparisons between alternative proteins and insect‐based options remain rare and the paucity of data regarding their environmental impacts makes these comparisons challenging. Water footprint, for instance, varies widely due in part to varying methodologies. In addition, some studies assume that ‘fresh’ insect biomass is equivalent to raw meat (Upcraft *et al*., 2021) while other studies consider a more valid comparison to be with processed insect products that mimic meat texture (Smetana *et al*., 2023*c*). Such disparities can be problematic, as processed products tend to have a larger environmental footprint (Lie‐Piang *et al*., 2021). For example, plant‐based meat substitutes have, on average, a 1.6–7 times higher environmental impact than less‐processed plant protein sources (e.g. pulses and peas) (Santo *et al*., 2020). Comparing ‘fresh’ insect biomass with alternative proteins might underestimate the environmental impact of the insect‐based products consumed.

### (5) Potential rebound effects

The net impact of promoting insect consumption compared to other environmentally conscious behaviours remains an open question. Some studies suggest that encouraging ‘green’ actions such as insect consumption can lead to unintended behavioural effects. For example, the moral licensing phenomenon involves people justifying less environmentally friendly actions due to their past positive behaviour (Burger, Schuler & Eberling, 2022). Encouraging such individuals to consume insects thus might inadvertently diminish their willingness to engage in other environmentally beneficial actions. Similarly, labelling insect‐based products as ‘sustainable’ might trigger the ‘negative footprint illusion’ (Gorissen & Weijters, 2016; Holmgren, Andersson & Sörqvist, 2018; Threadgold *et al*., 2021; Sörqvist & Holmgren, 2022), i.e. the belief that purchasing these ‘green’ products does not add to one's environmental footprint, potentially causing an increase in the overall consumption of these products. This effect was observed for insect burgers (Kusch & Fiebelkorn, 2019). However, given the complexity of consumer behaviour, further research is specifically needed on insect products. Future LCAs should consider rebound effects and evaluate their extent with quantitative calculations.

Since the environmental footprint of insect‐based foods tends to be higher than that of many plant‐based foods, discussions on entomophagy risk diverting attention away from more environmentally sustainable diets focused on plant‐based foods (Hodge, 2022). Shine (2020) questioned whether efforts and resources currently devoted to insect farming might be more effectively used to promote plant‐based foods, which are already familiar to consumers.

## V. ENVIRONMENTAL IMPACTS OF INSECT‐BASED FEED COMPARED TO CONVENTIONAL FEED

### (1) Overview of life cycle assessments

The most commonly used insect species for feed production are the BSFL, yellow mealworm, and, to a lesser extent, the common housefly (Huis, 2022; Gasco *et al*., 2023). The BSFL is versatile and can be fed a wide range of wastes, while the potential for using waste as a substrate for the yellow mealworm is more restricted (Le Féon *et al*., 2019; Harsányi *et al*., 2020; Quang Tran *et al*., 2022; Faes, 2022).

Table 3 summarises the environmental impacts of insect feed production compared to soybean meal, compound feed and fishmeal, which are among the most popular feed sources for aquaculture and chicken production. Studies focusing on the use of waste are discussed in Section IX.1. Note the limitations of the existing literature, including the small scale of studies, methodological differences, and variation in the use of insects as food, feed, or waste management solutions. Consequently, the environmental impact can vary by up to a hundredfold, depending on species, substrate, energy sources, methodology, scope and geographical location (Liverød, 2019; Smetana *et al*., 2023*c*).

**Table 3:** Environmental impacts of insect‐based products compared to common feed sources. Comparison between different life cycle assessments (LCAs), highlighting the context and limitations of each study, with data from commercial and pilot‐scale settings (excluding laboratory contexts). Studies that focus on the use of waste as a substrate are addressed in Section IX.1.

Study and location	Species	Greenhouse gas emissions (kg CO2e)	Land use (m^2^ per kg)	Water depletion (m^3^ per kg)	Energy use (MJ)	Context	Study limitations
**kg of dry matter**							
Reference values for soybean meal	NA	2.26 (average deforestation)^a^; 1.06 (no deforestation)^a^	0.062^a^ to 3.26^b^	0.04^b^	34.7 (average deforestation)^a^; 23.9 (no deforestation)^a^	kg of product	NA
Reference values for compound feed	NA	1.34^c^	1.48^c^	0.018^c^	5.81^c^	kg of product. Blend of cereals, oilseeds and other ingredients	NA
Reference values for fishmeal	NA	1.15^a^	0.005 ^a^ to 0.6–1.1^d^	0.35^e^	17.4^a^	kg of product	NA
Smetana *et al*. (2019) – Netherlands	*Hermetia illucens*	5.3	1.90	0.003	84.0	Industrial scale: more than 1000 tons dry larvae annually	High variability depending on the accounting method
Thévenot *et al*. (2018) – France	*Tenebrio molitor*	3.8^f^	4.1^f^	NA	141.3	Pilot: 17 tons larvae annually	Small scale, high energy use in processing
Roffeis *et al*. (2020) – Ghana	*H. illucens*	5.5	0.16	11.0	68.8	Small scale: 3.5–4.4 tons dry larvae annually	Small scale, use of chicken manure (in addition to brewery waste) as a substrate, warm country
Kleyn (2023) – South Africa	*H. illucens*	6.4	2.7	0.2	85.0	Small‐scale manufacturing: 52 tons of fresh insects annually	Small scale, carbon‐intensive electricity grid
Le Féon *et al*. (2019) – France	*T. molitor*	2.8	0.66	NA	59.2	Simulated system	Simulation based on other studies
Maiolo *et al*. (2020) – France	*H. illucens*	3.5	NA	4.71	283.9	Simulated system	Simulation based on other studies
Spykman *et al*. (2021) – various	*H. illucens*	5.0–11.0	0–8.0	−0.003 to 0.19	NA	Simulated system	Simulation based on other studies
**kg of wet matter**							
Suckling *et al*. (2020) – UK	*Gryllus bimaculatus*	21.1	NA	0.82	NA	Pilot: 12.5 tons wet insects annually for live pet food	Insects used for live pet food instead of conventional feed, small scale, cricket species, uncertainties on how much frass can be produced and used
**kg of dry protein**							
Reference values for soybean meal	NA	5.13 (average deforestation); 2.41 (no deforestation)	0.14^a^ to 7.4^b^	0.09^b^	78.9 (average deforestation)^a^; 54.3 (no deforestation)^a^	kg of protein^g^	NA
Reference values for fishmeal	NA	1.82^a^	0.008 ^a^ to 0.9 − 1.75^d^	0.55^e^	27.6^a^	kg of protein^g^	NA
Thévenot *et al*. (2018) – France	*T. molitor*	5.77^f^	6.35^f^	NA	217.4	Pilot: 17 tons larvae annually	Small scale, high energy use in processing
Defra & Ricardo (2024) – UK	*H. illucens*	30.2	NA	4.22	344.0	Uses data from other studies. Traditional wheat‐based feed scenario	Based on secondary data, although validated by an expert panel including three industry groups
Bosch *et al*. (2019) – Netherlands	*H. illucens*	3–19	3–67	NA	84–174	Range for 27 substrates. LCA using data from 40 other studies	Based on other studies
Spykman *et al*. (2021) – various	*H. illucens*	12–24	–1 to 18	−0.007 to 0.39	NA	Simulated system with 4608 production scenarios	Simulation based on other studies

NA denotes that a study did not include this variable in its LCA.

^a^ ECOALIM database (Wilfart *et al*., 2016). Version V8 (April 2023). Uses the land competition metric (m^2^) and provides average value for a kg imported into France from Peru (for fishmeal) and from Brazil (for soybean).

^b^ Ecoinvent 3 and Agrifootprint databases.

^c^ Smetana *et al*. (2023*a*) using data from the Agrifootprint database.

^d^ Samuel‐Fitwi *et al*. (2013).

^e^ Danish LCA Food Database.

^f^ Modahl & Brekke (2022) found 20% greater land use and CO2e per kg dry protein using data reported in these studies when using a lower nitrogen‐to‐protein conversion factor of 4.76 that is more appropriate for insects.

^g^ For protein‐normalised values, emissions per kg protein were calculated using reference data such as from the ECOALIM database, with mean protein contents of 44% for soybean meal and 63% for fishmeal (Thévenot *et al*., 2018).

Insect‐based feeds generally have higher CO_2_ emissions than soybean and fishmeal, with studies suggesting that GHG emissions for insect meals can range from 2.8 to 11 kg CO2e per kg dry matter, 21.1 kg CO2e per kg wet matter, and 3.0 to 30.2 kg CO2e per kg dry protein (Table 3). Some of the reported values apply only to specific contexts, such as the live pet food industry (Suckling *et al*., 2020). By contrast, soybean meal emissions average between 1.06 and 2.26 kg CO2e per kg dry matter, while fishmeal remains relatively low, averaging around 1.15 kg CO2e, according to the ECOALIM database.

Land use estimates also show large discrepancies, ranging from 0 to 8.0 m^2^ per kg dry matter. In comparison, soybean meal requires only 0.062–3.26 m^2^ per kg of product, depending on deforestation and the use of the land competition metric. Studies also report high energy use from insects, ranging from 59.2 to 283.9 MJ per kg dry matter (Smetana *et al*., 2023*a*). In comparison, production of 1 kg of soybean meal required 23.9–34.7 MJ, and 17.4 MJ for fishmeal.

Water use data for insect production are less consistently reported, but where data are available, they are variable, from as low as 0.003 m^3^ to 11 m^3^ per kg dry matter of product. Smetana *et al*. (2023*a*) calculated that insect farming generally has a higher water footprint than compound feed, with insects requiring 0.4–0.8 m^3^ of water per kg dry matter compared to 0.0179 m^3^ for compound feed. Methodologies for calculating water footprint are still evolving and may not always provide accurate results (Smetana *et al*., 2023*a*). The substrate for feeding insects was a major driver of water consumption, especially if insects were fed crop products (Miglietta *et al*., 2015; van Huis & Oonincx, 2017). The use of water for activities like mixing substrates, slaughtering insects, and maintaining facility hygiene can also be significant (Roffeis *et al*., 2020; Quang Tran *et al*., 2022).

Most studies appear to overestimate the protein content of BSFL, mealworms, and crickets (Janssen *et al*., 2017*b*; EFSA *et al*., 2024), leading to an underestimation of their environmental impact per kg of protein (Modahl & Brekke, 2022). This error has arisen because the standard nitrogen‐to‐protein conversion factor of 6.25 used for most foods is inappropriate for insects due to their non‐protein chitin content. For insects, the correct conversion factor is 4.76. When Modahl & Brekke (2022) applied this value to LCA calculations in Oonincx & de Boer (2012) and Thévenot *et al*. (2018), they reported 20% higher land use and CO_2_ emissions per kg dry protein compared to the original results.

### (2) Environmental impacts of insect meal as aquaculture feed

The use of insects as feed in aquaculture is a growing practice that is predicted to account for a significant portion of the insect market in the future. Aquaculture is a rapidly expanding market driven by a growing world population and demand for seafood. It accounted for 46% of seafood production in 2018 (FAO, 2020; Quang Tran *et al*., 2022). With forage fish stocks declining, finding environmentally sustainable feed options is a challenge for aquaculture, and insect farming has been presented as a solution (Froehlich *et al*., 2018; Jannathulla *et al*., 2019). Plant‐based feeds like soy meal are increasingly used as fish feed, but soy is linked with environmental impacts like deforestation, although efforts to source soy more sustainably are underway (Schilling‐Vacaflor & Gustafsson, 2024). Moreover, plant‐based feeds may not match the nutritional profile of fishmeal, resulting in lower aquaculture production yields (Silva *et al*., 2018). Nutritional aspects are important: insect‐derived feed ingredients can enhance the quality of farmed fish, a factor that mass‐based comparisons of environmental impacts may overlook (Liverød, 2019).

Quang Tran *et al*. (2022) conducted the latest systematic review of the environmental effects of insect aquafeed as a protein source. Overall, they found that while insect meals show benefits in terms of forage fish depletion (compared to fishmeal) and land use (compared to soy meal), these insect meals exerted an “enormous impact” on global warming potential, energy use, water consumption, acidification by nutrient pollution, and eutrophication (Quang Tran *et al*., 2022). Consequently, significant improvements are necessary to make insect meal a sustainable feed ingredient.

Incorporating insect meal into fish feed reduces the economic fish‐in fish‐out ratio (eFIFO) compared to fishmeal, reducing the pressure on marine resources. However, adding mealworms and BSFL to fish diet significantly increases faecal nitrogen waste production (Weththasinghe *et al*., 2021), a key contributor to eutrophication in aquatic ecosystems (Piedrahita, 2003; Amirkolaie, 2011), and potentially increasing ocean acidification (Quang Tran *et al*., 2022).

Subsequent studies have reached similar conclusions. A LCA in South Africa found that insect meal had a greater environmental impact than fishmeal across nearly all metrics, with CO_2_ emissions being 2–3 times higher (Kleyn, 2023). A LCA in Norway reported that while meal from BSFL reared on compost performed better than soymeal, BSFL reared using wheat bran and dairy waste as substrate – more representative of industry practices – resulted in GHG emissions twice as high (Zlaugotne *et al*., 2023). While BSFL had similar land use to soymeal, it consumed more energy and water. Yellow mealworm protein had an even worse environmental performance across these metrics (Zlaugotne *et al*., 2023). In a comparative LCA of aquaculture systems in Singapore, insect‐based meal had ‘higher environmental impacts than fishmeal and soybean meal for most impact categories’, even though the model ‘reduced electricity and water use to factor in technology optimization until 2040’ and assumed ‘a replacement of part of the feed by food waste’ (Bohnes & Laurent, 2021, pp. 6, 14).

A LCA focused on salmon farming found that switching from a fish‐based diet to an algal–insect diet resulted in a higher impact for most indicators, including climate change, resource use, energy use, terrestrial, marine and freshwater eutrophication, and acidification (Goglio *et al*., 2022). Biodiversity impacts were not assessed. It remains important to consider that these insect‐based products are still in their early development stages. Compared to poultry by‐product meal and microalgae, two other emerging aquafeed options, insects generally require less ‘emergy’, i.e. total energy investment, direct and indirect (Maiolo *et al*., 2021). Future innovations and scaling up of production, together with setting appropriate environmental targets, could potentially reduce their environmental impacts.

One study on aquaculture in Norway found more positive results for insect feeds (Modahl & Brekke, 2022). When insects were fed high‐value ingredients, such as grain or bran, their environmental impact was similar to that of conventional fish feed ingredients like soy, wheat, or faba beans. The authors suggested that the environmental impact of insect meal could be greatly reduced by using lower‐value feed ingredients, such as distiller's dried grains with solubles or cookie residues. In these cases, the environmental footprint of insect feed was similar to that of blue whiting protein, a type of fishmeal used in Norway. The different conclusions of this study are partly due to (*i*) their use of a CO_2_ emission estimate of 15 kg CO2e per kg dry protein for soybean meal, which is considerably higher than the 2.4–5.1 kg per kg dry protein reported in other research (see Table 3); and (*ii*) their economic modelling of side‐streams, with low‐value substrates being assigned lower environmental impact and potentially overlooking factors like the low nutritional value of waste leading to extended production cycles and increased energy consumption (Beyers *et al*., 2023).

Studies that find significant sustainability benefits for insect farming often focus on systems that utilise waste and non‐used side‐streams as feed substrates (Röthig *et al*., 2023). These studies also underscore the advantages of using locally sourced substrates, as transportation costs are a major contributor to GHG emissions in fishmeal production (Mertenat *et al*., 2019). The use of waste as insect feed is discussed in Section IX.1.

Alternative feed formulations may offer more positive outcomes. For example, one study designed eco‐diets for trout, incorporating changes like reducing fishmeal and fish oil by 50%, substituting soy meal with rapeseed meal, and using animal co‐products (Wilfart *et al*., 2023). These eco‐diets had lower environmental impacts across all categories compared to conventional diets, including reductions in GHG emissions (−46%), water dependence (−44%), and energy use (−42%). Growth rates were comparable in the short term, although probably lower in the long term. Although the authors considered whether to use insects in the eco‐diet, their inclusion was not pursued due to high costs and comparatively higher climate impacts.

In another comparative study, increasing fishmeal and fish oil production from trimmings and using marine fish in near‐shore sea cages were found to have a significantly lower impact than insect‐based meal (Bohnes & Laurent, 2021). Other strategies could help reduce the environmental impact of future fisheries by making fishmeal more sustainable. Properly managed fisheries work on maintaining stable fish stocks to ensure consistent yields over the long term. Successful examples of quota systems and total allowable catch strategies underscore the importance of effective management (Chu, 2009; Hoshino *et al*., 2020). Poorly managed fisheries will need to become well managed in the long term in every case (Hammer *et al*., 2010; van Gemert & Andersen, 2018).

The uncertainty that continues regarding their environmental and ecological impacts leads to caution towards endorsing insect‐based fish feed, especially as commercially viable alternatives exist. Due to several environmental concerns, the Global Animal Partnership's Atlantic salmon welfare standard, recognised as one of the most welfare‐comprehensive standards for the aquaculture sector, included a ban on insect‐based feed ingredients (Fletcher, 2022). The FAIRR Initiative, an investor network focused on environmental, social, and governance (ESG) risks and opportunities, also asserts that insects are ‘not a solution’ for sustainability in aquaculture (FAIRR, 2025).

### (3) Environmental impacts of insect meal as a livestock feed

Few studies have focused on the impacts of insects as chicken feed, although it is predicted to become the third largest portion of the insect market in the future (de Jong & Nikolik, 2021). Vauterin *et al*. (2021) assessed the potential of insect‐fed broiler chickens for meat production in Europe. Reviewing different LCAs and applying their results to broiler production, the study found that broiler chickens fed insects reared on grain‐based industrial feed would have higher GHG emissions than those fed soybeans (25.82 *versus* 18.50 kg CO2e per kg protein). Emissions varied widely, with maximum levels for insect‐fed chickens reaching 75.14 kg CO2e, and minimum values of 10.65 kg CO2e for chickens reared on waste‐fed insect meal. One limitation of this study was that it averaged data from a diverse range of studies and species, not limited to optimal scenarios, leading to some very high estimates of carbon emissions.

In comparison, pig feed is projected to be a fairly small part of the insect feed market (de Jong & Nikolik, 2021; Pexas & Kyriazakis, 2023). The impact of insect feed on ruminants, like cows or sheep, has not been explored, as these animals are not expected to become a major market for insect‐based feed (IPIFF, 2021; Ahmed & Nishida, 2023).

A more promising route to improving the sustainability of these food sources would be to improve the environmental sustainability of soybean production, such as adopting sourcing practices that exclusively involve soy cultivation on lands not recently subjected to deforestation. The EU has taken significant steps in this direction, such as the 2023 regulation for deforestation‐free supply chains (Regulation (EU) 2023/1115), mandating companies to confirm that products like soy are not linked to deforestation. Increasing domestic production of soy and maize in the EU is also a promising option (Ryba, 2024). Opting for certified soybeans from regions not associated with deforestation has been posited to result in a 47–53% reduction in the GHG emissions associated with soybean meal (Hörtenhuber *et al*., 2014; Wilfart *et al*., 2016; Vauterin *et al*., 2021). A transition towards sustainable soybean production holds the potential to decrease GHG emissions substantially, surpassing any potential impact of transitioning to insect‐based feeds.

## VI. ENVIRONMENTAL IMPACTS OF INSECT‐BASED PET FOOD COMPARED TO CONVENTIONAL PET FOOD

The pet food sector currently represents the largest market for insect proteins, accounting for about 50% of the total market of insects raised for food and feed (de Jong & Nikolik, 2021; Sogari *et al*., 2023*b*). Given its large size, minimising environmental impact is a critical concern in the insect pet food market. However, we only found one study (Bosch & Swanson, 2021) that explored in detail the environmental aspects of insect‐based pet food production. Several other papers do discuss this topic, but they typically compare the environmental impact of insects to meat rather than directly to pet food or meat co‐products (Bram, 2021; Schaap, 2021; Abd El‐Wahab *et al*., 2021; Duijnisveld & Myriam, 2022; Ahmed, İnal & Ri̇az, 2022; Valdés *et al*., 2022). Other studies briefly mention the potential of insects in pet food but lack detailed analysis. For instance Acuff *et al*. (2021) compare a range of pet food ingredients, showing that most (mainly animal by‐products) have a lower environmental footprint than insects. Several sustainability claims originate directly from the industry. Beynen (2018) reviewed 12 insect‐based pet food products and found that eight included a claim that insects are a sustainable protein source. Typically, the benchmark against which insect proteins were compared was human‐grade meat.

However, conventional protein sources in pet foods are often not human‐grade meat but meat co‐products like meat meals, organs, bones, and feathers (Pet Food Institute, 2020). These co‐products have a comparatively low environmental impact in a similar way to rearing insects on food waste. This makes pet food production “more sustainable than many human food processing industries in terms of cropland, energy, and water usage” (Acuff *et al*., 2021, p. 575). If insect meal is incorporated into pet food, it is likely to replace these meat co‐products, which are not farmed explicitly for this purpose and have low economic value. While some studies suggest that pet food has a high environmental impact (Okin, 2017; Su, Martens & Enders‐Slegers, 2018), they often incorrectly assume that meat is the primary protein source. Moreover they “do not provide reference data on the impact of these conventional pet food ingredients”, complicating direct comparisons (Bosch & Swanson, 2021).

Bosch & Swanson (2021) calculated that, on average, insect proteins for pet food emit 2–10 times more GHGs than conventional pet food products. They refer to a Blonk Consultants report, which estimated the carbon footprint of pet food at ‘about 1 kg CO2e per kg protein for a mixed meal and 2 kg per kg protein for a poultry meal’ (Bosch & Swanson, 2021, p. 802). In comparison, emissions from insect production are higher, ranging from 3 to 30.2 kg CO2e per kg dry protein (see Table 3).

An interesting case in France involved the company Tomojo, which faced scrutiny over its marketing claims about the environmental benefits of its pet food. The company advertised its products with assertions such as ‘Sustainable proteins approved by the planet’ and ‘For an ecological diet’, comparing the impact of insects with beef production rather than with meat co‐products. An investigation by the French Advertising Standards Jury (Jury de Déontologie Publicitaire, 2021) deemed the claims unjustified and misleading.

Additionally, comparing insect‐based sources with other alternatives is essential. Plant‐based pet foods are sometimes estimated to have a lower carbon footprint than animal‐based ones (Acuff *et al*., 2021). The vegan pet food market, valued at $8.6 billion in 2021, is growing and is projected to reach $15 billion by 2028 (The Insight Partners, undated). Regarding health, while there are numerous methodological limitations with the existing literature, the latest systematic review found that plant‐based pet foods are comparable, or perhaps slightly more advantageous, for the health of pet dogs and cats (Domínguez‐Oliva *et al*., 2023). However, a cautious approach is warranted, as further validation and controlled clinical trials are required (Davies, 2022). Important uncertainties remain, but the same is true for insect diets: data are limited on the nutritional quality and digestibility of insects (Bosch *et al*., 2014; McCusker *et al*., 2014; Mouithys‐Mickalad *et al*., 2020; Acuff *et al*., 2021).

## VII. ENVIRONMENTAL IMPACTS OF FRASS

‘Frass’ refers herein to the residue left by insect farming, consisting of excrement, leftover substrate, and insect body parts (European Commission, 2021). For BSFL, frass can represent over a third of the original substrate's mass (Basri *et al*., 2022). If insect production grows significantly, it will generate high quantities of frass, which will need to be managed in an efficient and sustainable way (Gebremikael *et al*., 2020; Houben, Daoulas & Dulaurent, 2021; Watson, Houben & Wichern, 2022).

The insect industry has proposed using frass as a fertiliser (Basri *et al*., 2022), with most relevant data coming from the EU. The use of frass is central to claims that insects can contribute to a circular economy, allowing the recirculation of nutrients (Poveda, 2021). Its application could potentially help offset the environmental impacts associated with conventional fertilisers, which include high energy and resource consumption, and pollution leading to eutrophication and soil acidification (Savci, 2012; Schmitt & de Vries, 2020; Chojnacka, Moustakas & Witek‐Krowiak, 2020). While there are suggestions of using frass as biochar, animal feed or feedstock, there are fewer data on these applications (Basri *et al*., 2022), and using frass as animal feed is prohibited in the UK, USA and EU.

To date, the use of frass as a fertiliser is very limited (Jasso *et al*., 2024). A complicating factor is the EU's required disinfection process by which all frass must be treated at ≥70 °C for 1 h to avoid contamination. Heating the frass has some undesirable effects, such as killing beneficial microbiota and destroying biomolecules that enrich soils (Poveda, 2021; Zunzunegui *et al*., 2024). However, contamination is a serious issue (Food and Agriculture Organization, 2021). When insects are fed with waste, there is a risk that frass could contain pathogenic microorganisms (Basri *et al*., 2022). A recent review identified high levels of contamination in larvae and frass across various substrates (including cereals, fruits, vegetables, and agri‐food co‐products) with pathogens such as *Salmonella* spp., *Xanthomonadaceae*, *Staphylococcus aureus*, *Clostridium perfringens*, *Escherichia coli*, and *Bacillus cereus* (Wynants *et al*., 2019; Kawasaki *et al*., 2020; Brulé *et al*., 2024). Sterilising the substrate has been shown to impact the productivity of BSFL rearing and may undermine the benefits of frass when used as a fertiliser (Gold *et al*., 2020; Siddiqui *et al*., 2024*a*). However, few studies have been performed on treated frass (Zunzunegui *et al*., 2024).

Market growth is hindered by regulatory constraints, such as the requirements for heat treatment and limits on the inclusion of insect body parts and eggs in frass (Eurogroup for animals, 2023). The insect industry is currently lobbying to reduce or remove some requirements, such as heat treatment, suggesting that without these changes the use of frass as a fertiliser is not economically viable under current regulations. The removal of heat treatment raises concerns about the ecological and health implications of spreading untreated insect waste in the environment (Poveda, 2021; Basri *et al*., 2022).

Frass contains high quantities of both macro‐ and micronutrients – especially nitrogen, phosphorus, and potassium – offering advantages over synthetic fertilisers that typically supply only macronutrients (Houben *et al*., 2020; Watson *et al*., 2021*b*; Jasso *et al*., 2024; Siddiqui *et al*., 2024*a*; Zunzunegui *et al*., 2024). Antimicrobial peptides naturally present in BSFL can act as a defensive barrier for the plant (Basri *et al*., 2022). Containing beneficial microbes, frass also can enhance plant resilience to stressors like flooding and disease, acting as a form of biological pest control (Poveda, 2021; Barragán‐Fonseca *et al*., 2022; Beesigamukama *et al*., 2023). For instance, the chitin in *T. molitor* frass triggers defences against Fusarium wilt disease (Quilliam *et al*., 2020). Frass also contains nitrogen‐fixing bacteria that increase plant nitrogen uptake, promoting plant growth (Siddiqui *et al*., 2024*a*). Components of frass are readily absorbed by plant roots, it adds carbon, nitrogen, and ammonium to the soil and decomposes faster than synthetic fertilisers, enhancing soil quality (Houben *et al*., 2020; Poveda, 2021; Jasso *et al*., 2024).

Frass resembles chicken manure in composition, but due to the diverse substrates used in insect farming and lack of standardisation, it can show considerable variability both in its nutritional composition and microbial diversity, resulting in environmental impacts that can vary widely and nutrients that may not meet the nutritional requirements of particular crops (Schmitt & de Vries, 2020; Gebremikael *et al*., 2020; Poveda, 2021; Zunzunegui *et al*., 2024). Further research will be necessary to understand fully the capacity of frass to enhance crop productivity and soil health, identify optimal characteristics and inclusion levels, and identify whether frass can be a comprehensive replacement for organic fertiliser (Bloukounon‐Goubalan *et al*., 2021; Jasso *et al*., 2024; Zunzunegui *et al*., 2024). Although frass has been used in high‐value woody crops or horticultural crops, currently “its use in extensive crops is far from being possible” due to a lack of research (Zunzunegui *et al*., 2024, p. 8).

Some studies note negative effects on soil processes, such as excessive nitrite accumulation in the soil (Watson, Preißing & Wichern, 2021*a*) or inhibited seed germination (Kawasaki *et al*., 2020). While several studies indicate that frass can increase yield, others reported negative growth associated with plausible phytotoxicity of the frass (Kagata & Ohgushi, 2012; Alattar, Alattar & Popa, 2016; Berggren *et al*., 2019; Lopes, Yong & Lalander, 2022). High moisture content in substrates used for BSFL rearing, such as food waste, often leads to immature, wet frass with high ammonium levels and low porosity, causing ammonia poisoning that hinders plant growth, and complicates processing and handling (Alattar *et al*., 2016; Cheng, Chiu & Lo, 2017; Lalander *et al*., 2020; Siddiqui *et al*., 2024*a*). However, reducing the moisture content could lead to slower BSFL growth (Siddiqui *et al*., 2024*a*). BSFL frass can lack optimal nutrient availability for promoting robust plant growth, especially for supporting extensive root development, which reduces a plant's ability to access nutrients from deeper soil layers, potentially impacting overall growth and yield (Gebremikael *et al*., 2022).

There is a lack of research on the impacts of frass on the environment, especially as most existing data are from laboratory studies with limited insights into long‐term impacts (Siddiqui *et al*., 2024*a*). The use of frass would lower the environmental impacts of rearing insects as food and feed by providing an additional product (Siegrist *et al*., 2023). Some studies indicate that using frass can reduce CO_2_ emissions by 12–16% compared to the use of mineral fertilisers (Thévenot *et al*., 2018; Modahl & Brekke, 2022), and may lower emissions of CO_2_, NH_3_, CH_4_, and N_2_O compared to compost (Pang *et al*., 2020; Song *et al*., 2021).

Smetana *et al*. (2019) found superior results for insect frass over other organic fertilisers, including a reduction in both aquatic and terrestrial acidification (with decreases of 0.064 g and 0.265 g of SO_2_ equivalents per kilogram of frass used, respectively). However, Schmitt & de Vries (2020) “nuanced this conclusion by suggesting that environmental impacts need to use comparable fertilizing units as a baseline”, while “the macronutrient, micronutrient and pathogen contents, as well as the GHGs produced during the process, are highly dependent on the inputs used to produce the fertiliser and the amendment” (Hénault‐Ethier *et al*., 2024, p. 5). Meanwhile, it “remains to be seen whether insect frass… has a lower environmental footprint than conventional farm manures” (Hénault‐Ethier *et al*., 2024, p. 6). A study on organic liquid fertiliser derived from waste‐fed insect frass found GHG emissions to range from six times higher to four times lower than conventional fertilizers, depending on nitrogen losses (Desaulniers Brousseau *et al*., 2024).

Another key concern is that the stimulatory effects of frass on the soil may have negative environmental impacts, which have been largely overlooked. Several studies have reported significant GHG emissions from soils amended with frass (Gebremikael *et al*., 2020; Houben *et al*., 2021; Rummel *et al*., 2021; Watson *et al*., 2022; Beesigamukama *et al*., 2023). One study demonstrated that due to an increase in basal respiration, soils treated with frass emitted considerably more CO_2_ than those treated with conventional compost or left unfertilised (Fuhrmann *et al*., 2022). Another study found that frass altered soil microbial composition, changing nutrient fluxes and leading to substantial carbon and nitrogen releases (as CO_2_, CH_4_ and N_2_O) (Rummel *et al*., 2021). According to the authors, “very high” GHG emissions were reported, “undermining the potential environmental benefit of insect‐based protein production and calling for more detailed analyses before frass is widely applied in agriculture” (Rummel *et al*., 2021, p. 1).

An open question is whether frass can compete economically with more traditional fertilisers. In Australia, the current price of frass is significantly above what farmers are willing to pay (Kragt, Dempster & Subroy, 2023): it currently costs between $1,500 and $3,000 per tonne depending on specifications, significantly higher than compost and manure at $300–$350 per tonne (Kragt *et al*., 2023). In the EU, frass received authorisation as a fertiliser in November 2021 (Commission regulation 2021/1925). Despite this, the frass market faces significant competition from organic fertilisers, particularly livestock manure, which already saturates the EU fertiliser market (Ffoulkes *et al*., 2021) as more manure is generated than is used as a fertiliser (Cox, 2019). As a result, some insect producers have resorted to exporting their frass abroad as a means of disposal (Ffoulkes *et al*., 2021).

The market for organic fertilisers is smaller than that for chemical fertilisers and poses additional challenges. Organic fertilisers, including frass, offer environmental advantages but often require more labour and financial investment (Wang *et al*., 2018), and tend to be costlier to transport over long distances. These issues raise doubts about the capacity of insect frass to reduce the usage of chemical fertilisers substantially.

If insect waste is not revalorised, it will need to be disposed of. In that case, the massive amount of frass generated by farming insects may become a serious environmental problem (Poveda, 2021). Only a limited amount of material can be stored onsite as frass can become hazardous if not disposed of or utilised promptly (Ffoulkes *et al*., 2021). Reports indicate that already existing struggles with maintaining large volumes of conventional livestock manure lead some farmers to resort to illegal disposal methods (Wasley *et al*., 2017; Cox, 2019). Such environmental crime represents a threat to ecosystems and biodiversity due to eutrophication (Neve, 2023, p. 52). Managing insect farm waste then could compound the environmental issues already associated with traditional aquaculture and livestock production, particularly regarding air and water pollution (European Food Safety Authority, 2015; Halloran *et al*., 2016).

A review of 50 highly cited studies commissioned by the UK government, aimed at assessing the environmental potential of insect farming, concluded that “the usefulness and safety of frass being spread in large quantities over farmland is unknown” (Defra & Ricardo, 2024; summarised in Ricardo, 2025, p. 1). They highlighted a lack of consensus on application rates, missing field trials, insufficient standardisation and a lack of understanding of the impacts on soil biology, nitrogen, and soil GHG emission. More data are needed to understand fully the wider impacts of frass before it can be considered a viable contributor to a circular economy (Watson *et al*., 2022).

## VIII. IMPACTS ON BIODIVERSITY AND ZOONOTIC DISEASES

### (1) Biodiversity threats and invasive species

One environmental issue shared by use of insects as food or feed concerns impacts on local biodiversity. Farmed insects, if released into natural environments, could pose risks by adversely affecting local insect populations, potentially disrupting local natural ecosystems through competition with native species or by introducing harmful genes into wild populations (Yen, 2015; Halloran *et al*., 2018; Wilderspin & Halloran, 2018; Lourenço *et al*., 2022; Siddiqui *et al*., 2024*b*). Research indicates that genes selected for farmed colonies have already been transferred to wild BSFL populations in Europe (Generalovic *et al*., 2023). This precaution is less relevant to yellow mealworms which are widely found in stored goods, where they are considered a grain pest, although they are considered invasive in Moldova (Lourenço *et al*., 2022).

Escapes could occur during natural disasters or other unforeseen events, as seen for livestock in the US during Hurricane Florence (Graff, 2018). An additional challenge for insect farming, unlike conventional livestock, is the near‐impossibility of recapture. Weissman *et al*. (2012, p. 79) argue that if commercial cricket species are approved for import, we should “expect them to be introduced into the environment whether through accidental escape or intentional release”. Even in high‐income countries, ‘the biosecurity status of these rearing facilities is worrying’, with a ‘frequent and high numbers of escapees’ and a lack of regulatory policy guidelines (Bang & Courchamp, 2021, p. 395). This is the case even for globalised species that are not considered invasive, like the BSFL. In another concerning example, an examination of insect‐based protein bars purchased online revealed that some contained larval‐stage insect pests, which could contribute to the spread of invasive species (Giusti *et al*., 2024).

Past instances of invasive insect species include Africanised bees, commonly known as ‘killer bees’, and the spongy moth (*Lymantria dispar dispar* L.). Africanised bees originate from East African lowland honey bees (*Apis mellifera scutellata* L.) brought to Brazil for a cross‐breeding experiment with European honey bees to boost honey production (Smithsonian Institution, undated). However, in 1957, a mishap led to the escape of 26 selectively bred queen bees and their workers (Winston, 1992), resulting in the spread of hybrids to other South American nations, Central America, Mexico, and the USA. Similarly, spongy moths were brought to the USA by a single individual aiming to crossbreed them with silk moths for the silk industry (Doane & McManus, 1981). These moths have become a significant threat to North American forests, damaging trees through defoliation (USDA, undated). Their economic impact is substantial: by affecting tourism and generating costs for tree removal and replacement, they lowered residential property values by an estimated 120 million USD annually in the US from 1998 to 2007, while federal expenditures for controlling the spongy moth reached 298 million USD over the same period (Invasive Species Centre, 2019).

In the EU, risk assessments have been conducted prior to authorising farming of additional insect species; however, these assessments have primarily taken place in northern countries, with risk evaluation for southern regions largely missing (Lourenço *et al*., 2022). While some species, like the black soldier fly, were initially considered unlikely to establish in the wild (Spranghers *et al*., 2017), more recent evidence has reached a contrasting conclusion (Roháček & Hora, 2013; Jonsell, 2017). So far, gene mixing between domesticated and wild BSFL populations is not widespread in non‐native areas. However, near BSFL farms and research centres, increased mixing may disrupt local genetic adaptations, posing a threat to native populations. More competitive domesticated strains could invade new and existing habitats due to human activities (Kaya *et al*., 2021). Experts reporting to the European Commission highlight that these risks should not be discounted and that the precautionary principle should be exercised, especially given the short lifespans and rapid rates of dispersal of these insects (EU Platform on Sustainable Finance, 2021).

High‐density insect farms expose insects to various diseases and pathogens, including novel strains (Weissman *et al*., 2012; Jansson, Hunter & Berggren, 2019). This raises concerns about escaped insects transmitting these diseases to wild populations, especially pollinators, which are already facing numerous threats. The impact of diseases such as the densovirus that devastated the American cricket pet food industry highlights the potential risks to local biodiversity (Weissman *et al*., 2012; Jansson *et al*., 2019). In response to this disease, cricket producers' search for a virus‐resistant cricket species inadvertently led to the distribution of a previously unnamed *Gryllus* species across Europe and the USA, posing potential risks to native fauna and agriculture. It is also likely that destructive pathogens originating from commercial bumblebees have been ‘spilling over’ into wild bee populations throughout North America (Otterstatter & Thomson, 2008). The topic of diseases in insect farming is addressed in more detail in Section VIII.2.

The introduction of genetically modified insects, bred for enhanced size, strength, growth rate, adaptability, and resilience, could multiply concerns about invasive species (Moccia, 2022). Research is already underway to produce improved insect strains using genetic editing and selection (Huis, 2022). Selectively bred species could have undesirable phenotypes that could lead to genetic pollution – the spread of contaminated altered genes to natural insect populations, potentially reducing their fitness (Ellstrand, 2001). This is a known problem in other types of animal agriculture, such as aquaculture, as seen with the detrimental effects on wild fish populations following the escape of farmed fish. There are several cases where farmed salmon, mostly products of selective breeding (Janssen *et al*., 2017*a*), reproduced with wild populations. This led to the transmission of altered genetic characteristics in wild populations, with lower lifespans, reduced individual fitness, and increased vulnerability to diseases (Glover *et al*., 2017; Faust *et al*., 2018).

One potential risk‐management strategy could involve genetically modifying insects to prevent their spread in the wild. Extensive research has been conducted on this topic to control pest populations, such as disease‐carrying mosquitoes or moths, by reducing their reproductive capacity or making them infertile (Waltz, 2017; Devos *et al*., 2022). Research on the use of this strategy in farmed insects is limited, highlighting the need for further studies and appropriate regulatory frameworks.

Jansson *et al*. (2019) propose a conservative approach to insect farming in Sweden, recommending the exclusion of non‐native species in food and feed production systems. This stance, rooted in the precautionary principle, is supported by Berggren *et al*. (2019), who advocate for the use of non‐native species only when substantiated by robust scientific evidence of safety.

This restrictive guideline has several implications for the insect farming industry. The limitation on available species may constrain producers' ability to optimise efficiency, potentially increasing the environmental footprint of insect farming operations. Such restrictions also could have significant economic ramifications, potentially hampering industry growth and competitiveness. Implementation of these constraints would necessitate the development of a dedicated legal structure, likely impeding industry growth. Without compensatory measures, such as targeted incentives or penalties on established industries, these constraints might impede the dissemination of innovations within the sector. This cautious approach, while aiming to safeguard ecological integrity, results in a complex trade‐off between environmental protection and industry development.

### (2) Zoonotic diseases and antibiotic use

Another topic concerns disease management. The literature suggests that, compared to birds and mammals, edible insects present a relatively low risk of transmitting zoonotic diseases to humans, primarily due to taxonomic distance (Doi, Gałęcki & Mulia, 2021; Lange & Nakamura, 2021; Gałęcki, Bakuła & Gołaszewski, 2023). There is only a small number of reported pathogens detected in the black soldier fly to date (Joosten *et al*., 2020; Huis, 2022). Furthermore, the controlled conditions of insect farming can help to reduce pathogen spread (Faes, 2022). There are significant health concerns with conventional livestock that could be mitigated by the use of insects as a meat replacement (Doi *et al*., 2021). On the other hand, the lack of reported pathogens may simply be due to low research effort rather than a genuine lack of pathogens, as recent scientific studies and anecdotal evidence from scientists working with black soldier flies support the suggestion that the number of pathogens may be higher than originally thought (InsectDoctors, 2023; She *et al*., 2023).

Nevertheless, insects are not completely free from pathogens that could impact human health (Berggren *et al*., 2019). At least one study has suggested that viruses associated with insect production could pose a risk to both human and animal health (Bertola & Mutinelli, 2021). Insects can be the primary or intermediate hosts or carriers of human diseases (Marshall, Dickson & Nguyen, 2016; Jansson *et al*., 2019; Faes, 2022). For example, mealworms have been identified as a potential disease vector in poultry (Rumbos *et al*., 2019). While viruses pathogenic in vertebrates cannot replicate in insects, they can still transmit them passively, acting as a vector (Doi *et al*., 2021). As the microbiological safety of edible insects is still under debate (Gałęcki *et al*., 2023), appropriate sanitary and biosecurity rules should be applied (Doi *et al*., 2021). The potential for insects to transmit harmful pathogens to humans has not been explored sufficiently and requires further investigation (Berggren *et al*., 2019; Lange & Nakamura, 2021; Bertola & Mutinelli, 2021; Aidoo *et al*., 2023).

Edible insects may be an ‘underestimated reservoir of human and animal parasites’ and potentially ‘the most important parasite vector for domestic insectivorous animals’ (Gałęcki & Sokół, 2019, p. 1). A study of small‐scale insect farms for pet food found parasites in over 80% of them. In 30% and 35% of these farms, these parasites had the potential to affect humans and animals, respectively. These parasites can play a role in the dispersion of invasive diseases (van der Fels‐Klerx *et al*., 2018; Doi *et al*., 2021; Gałęcki *et al*., 2023).

Pathogen outbreaks can devastate insect populations, posing production risks (Huis, 2022). In the event of diseases, entire insect populations in farms may need to be eradicated. The future of disease management in insect farming remains uncertain (Maciel‐Vergara & Ros, 2017; Berggren *et al*., 2019), although in a recent survey of industry stakeholders, this issue is ‘considered of medium concern relative to other “operational” barriers’ (Niyonsaba *et al*., 2023, p. 700). The use of antibiotics in insect farming remains uncertain as it is unclear whether this would be effective or desirable, considering the risk of antimicrobial resistance (Suckling *et al*., 2020). Nonetheless, initial antibiotic use in insect farming has been low (Halloran *et al*., 2016), and the industry claims that they are not used (IPIFF, 2020*b*), which could help mitigate antimicrobial resistance risks if insects act as meat substitutes.

However, it remains unclear whether this is likely to remain this way. Intensive farming of insects might face similar pressures as other animal farming industries, where intensification is a key factor in disease emergence (Slingenbergh *et al*., 2004; Jones *et al*., 2013; Lange & Nakamura, 2021). Blanket treatments in response to disease can lead to antimicrobial resistance, reducing the effectiveness of antimicrobials over time. This scenario has been evidenced in diverse animal farming industries, such as in pig or salmon farming, where novel zoonoses emerged followed by antimicrobial resistance. The use of antibiotics has become frequent even in shrimps, another arthropod group (Holmström *et al*., 2003; Halloran *et al*., 2016), and in silkworms, one of the most commonly farmed insects (Li *et al*., 2020). Some studies indicate that insects represent a reservoir for antibiotic‐resistant bacteria (Zurek & Ghosh, 2014; van der Fels‐Klerx *et al*., 2018). As mentioned by the Food Standards Agency (2022, p. 30), ‘there is a potential hazard that the rearing of edible insects on a large scale may incur the use of antibiotics […], contributing to AMR [antimicrobial resistance]. The exact impact of this practice is not possible to determine with the available information’.

## IX. FUTURE POSSIBLE IMPROVEMENTS

### (1) Environmental impacts of feeding insects with waste

The utilisation of food waste as a substrate for insect farming is frequently suggested as a prospective solution to improve their environmental impacts and enhance the circularity of the food system (Madau *et al*., 2020; Rehman *et al*., 2023).

It is noteworthy that most Western insect farms make little use of food waste, partly due to regulatory constraints that prohibit the use of certain proposed waste products on the grounds of public health regulations, as well as logistical and economic constraints (Salemdeeb *et al*., 2017; Skrivervik, 2020; Ffoulkes *et al*., 2021; Sillman, 2021; Mancini *et al*., 2022; Fischer, 2022; Biteau *et al*., 2024*b*). Some insect species, like crickets and yellow mealworms, have limited potential to be fed on household waste (Le Féon *et al*., 2019; Harsányi *et al*., 2020; Quang Tran *et al*., 2022; Faes, 2022). They perform better on high‐quality feed such as crop by‐products, but these can often serve as livestock feed. By contrast, BSFL are more adaptable and capable of consuming a broader array of food waste.

Some substrates discussed below have not received regulatory approval. In the UK and EU, permitted waste products and by‐products are limited to processing waste and former foodstuffs consisting solely of vegetal, dairy, egg, and/or honey origins, as laid out in the EU Regulation 2022/1104. In the US, BSFL, the sole insect authorised for animal feed, must be “raised on a feedstock composed exclusively of feed‐grade materials” (Association of American Feed Control Officials, 2021, p. 1). Due to safety concerns, substrates incorporating manure or mixed waste materials are restricted. These substrates are mentioned here in the context of potential future improvements, although they may not receive regulatory approval in the foreseeable future.

Broadly speaking, feeding insects with wastes, food processing by‐products, or manure rather than commercial grain‐based co‐products tends to reduce the environmental impact of the resulting insect‐derived products (van Zanten *et al*., 2015; Smetana *et al*., 2016,2021*b*; Salomone *et al*., 2017; Roffeis *et al*., 2017; Bosch *et al*., 2019; Ites *et al*., 2020). However, the scarcity of studies and the lack of industrial‐scale research make it challenging to predict exactly by how much. A review focusing on BSFL found a lack of data on emissions, and that the absence of robust guidelines and protocols complicates comparisons across studies (Van Peer *et al*., 2022). For instance, while rearing conditions significantly affect emissions, the specific details are often inadequately reported, and nearly every experiment employs a different protocol (Deruytter *et al*., 2022). GHG emission estimates for BSFL farming differ by a factor of up to 12 among studies (Jensen *et al*., 2021).

The use of food waste as feed can significantly change GHG emissions, with estimates ranging from a beneficial −6.42 to 5.3 kg CO2e per kg of insect meal (Smetana *et al*., 2019; Bosch *et al*., 2019; Ites *et al*., 2020; Boakye‐Yiadom, Ilari & Duca, 2022; Pahmeyer *et al*., 2022; Johansson, 2023; Elsayed *et al*., 2024). A negative value arises where feeding food waste to insects helps avoid the need for more costly waste‐treatment methods. Typically, waste is managed through processes like landfill or incineration, which carry associated environmental impacts. When insects are used to process waste, they reduce the necessity for these treatments and their related environmental impacts.

Feeding insects with different waste materials yields different environmental outcomes (Quang Tran *et al*., 2022). For instance, feeding BSFL with brewery grains can have a positive environmental impact, whereas feeding them on potato peelings has a negative impact (Ites *et al*., 2020). Using expired food appeared to offer no significant savings compared to standard waste treatment. The suboptimal environmental outcome associated with using potato peelings and expired food can be attributed to reduced efficiency of rearing insects on these substrates, leading to extended growth periods and lower productivity (Spykman *et al*., 2021). In some studies, BSFL fed on cattle manure and municipal waste had a better environmental impact than when using traditional animal feeds such as beet pulp (Smetana *et al*., 2016). However, these waste‐fed BSFL had similar impacts compared to other animal feeds such as distiller's grains with solubles.

Rearing insects on waste can result in improvements to certain environmental metrics while negatively impacting others. A German study found that a trout feed diet composed of three‐quarters waste‐fed BSFL used 80% less water and 90% less land, but required 50% more energy and produced five times the GHG emissions compared to a conventional trout feed (mainly fish meal, fish oil, and soy meal) (Goyal *et al*., 2021). Similarly, an LCA commissioned by the UK government, validated by three industry groups, found that BSFL fed on waste from restaurant kitchens or chicken manure performed worse than soybean meal on 14 out of 16 environmental indicators, emitting at least 5.7 times more GHG emissions (Defra & Ricardo, 2024). BSFL also underperformed relative to fishmeal, performing worse on 10 out of 16 indicators and emitting 1.8 times more GHG emissions.

While feeding insects with manure provides potential environmental benefits, a review of 75 BSFL production systems revealed contradictory results, with outcomes varying significantly based on factors like BSFL strain (Grassauer, Ferdous & Pelletier, 2023). Additionally, since most of these systems are micro‐scale (i.e. in laboratory settings), they provide only limited insights for large‐scale production. For example, impacts ranged from 0.77 to 12 kg CO2e per kg dried insects (Roffeis *et al*., 2017) and 1 to 7 kg CO2e per kg of proteins (Bosch *et al*., 2019). Another study found no clear answer to whether composting pig manure is better with or without insects, as the environmental impacts varied depending on the specific impact category assessed (Beyers *et al*., 2023). When insects are reared on manure, ammonia and methane emissions can be considerable (Van Peer *et al*., 2022).

Compared to traditional waste‐treatment methods (landfill, incineration, composting, biogas and bioconversion plants), utilising waste as feed for BSFL has shown encouraging results on several metrics, although anaerobic digestion can outperform BSFL on global warming potential and energy consumption (Mondello *et al*., 2017; Mertenat *et al*., 2019; Ites *et al*., 2020; Kim *et al*., 2021; Nugroho *et al*., 2023; Ferronato *et al*., 2024). Emissions from treating a ton of biowaste with insects range widely, from −432 kg CO2e (indicating a net benefit) to 877 kg CO2e, with an average of 70 kg CO2e across 12 LCAs (Salomone *et al*., 2017; Smetana *et al*., 2019; Boakye‐Yiadom *et al*., 2022), and insects do not always surpass traditional waste treatment, animal feed, or biodiesel options (Frasnetti, Sadeqi & Lamastra, 2023). This variability stems from differences in system boundaries; studies reporting low emissions often exclude processes like substrate collection, larvae production, pre‐treatment and transport (Komakech *et al*., 2015; Guo *et al*., 2021), whereas higher values (up to 877 kg CO2e) account for facility construction, frass processing and larvae production (Smetana *et al*., 2019; Boakye‐Yiadom *et al*., 2022).

Some studies have shown that including the transportation of feed substrates in a LCA can increase CO_2_ emissions and energy consumption by up to 67% (Liverød, 2019; Ites *et al*., 2020). Researchers advise that the substrate's geographical origin therefore is important for insect production's environmental and socioeconomic performance (Roffeis *et al*., 2020; Ferronato *et al*., 2024), and that locally available by‐product streams should be preferred (Derler *et al*., 2021; Adamaki‐Sotiraki *et al*., 2024). However, other findings indicate that transport may represent only a minor component, accounting for less than 4% of total emissions (Modahl & Brekke, 2022).

Ultimately, the environmental benefits of using BSFL as a waste‐to‐feed solution depend on the specific feed source and its origin. The positive outcomes observed for certain feed types cannot be generalised to all waste materials or insect species without thorough evaluation (Ites *et al*., 2020; Modahl & Brekke, 2022; Athanassiou *et al*., 2024).

Furthermore, conventional livestock can also consume some waste products. Nearly 30% of global livestock feed intake consists of agricultural co‐products, byproducts, and food‐processing residuals (Mottet *et al*., 2017; Dou, Toth & Westendorf, 2018; McBride *et al*., 2021; Food Standards Agency, 2023). Food waste from the hospitality sector and households and surplus products from bakeries and confectioneries could serve as protein sources in livestock diets (Pinotti *et al*., 2021; Food Standards Agency, 2023) with lower environmental impacts than anaerobic digestion (Shurson, 2020). Notably, some substrates appropriate for insect nutrition are already widely used as livestock feed, including brewery grains, spent grains, and distiller grains with solubles. Consequently, the range of waste exclusively suitable for insect consumption is narrower than usually assumed. Given this, using waste‐fed insects as animal feed would be ‘inherently less efficient’ than feeding food waste to animals directly (Salemdeeb *et al*., 2017; Verkuijl *et al*., 2024). By competing with products that would otherwise be used as pig or poultry feed, insect farming may even intensify pressures on arable land (Verkuijl *et al*., 2024).

Certain waste types, like manure, are unsuitable for feeding to conventional livestock and can be ingested by some insect species, but also have inherent drawbacks (e.g. higher mortality rates) and obvious health concerns. Despite the common assumption that it is more sustainable to feed insects materials unsuitable for conventional livestock (Smetana *et al*., 2019; Modahl & Brekke, 2022), this may not always hold true (Spykman *et al*., 2021). One LCA found that producing BSFL meal from non‐utilised side streams that are typically reincorporated into fields or left to decompose had environmental impacts 10–100 times greater than using soybean meal or fishmeal in terms of GHG emissions, water use, and energy consumption (Beyers *et al*., 2023). While the residues themselves had a low environmental impact, their limited nutritional value delayed larval maturation, extending the production cycle and increasing energy use; they also required more feed and yielded a less favourable nutritional profile (Spykman *et al*., 2021). To achieve suitable nutritional levels, it was necessary to supplement the residues with higher‐value agricultural products.

Comparative studies on the environmental impacts of waste‐fed insects *versus* waste‐fed farm animals are scarce. A study by the World Wide Fund for Nature (WWF) assessed three food‐waste‐to‐feed pathways for egg production: food waste converted into BSFL meal for hens, processed food waste fed directly to hens, and bakery by‐products fed to hens (McBride *et al*., 2021). Results were mixed (Table 4), with BSFL meal having significantly higher impacts than the baseline in terms of global warming potential and water consumption, but requiring less land. While using renewable energy reduced carbon footprints by 0.1–51%, BSFL‐based diets still had higher GHG emissions than the baseline and other food waste‐based diets.

**Table 4:** Comparison of environmental impacts of three food‐waste‐to‐feed pathways for egg production, in which hens were fed either black soldier fly larvae (BSFL) meal from BSFL reared on food waste, processed food waste feed, or bakery by‐products meal at an inclusion level of either 5, 10, or 15%. Results are expressed relative to a baseline diet (100%). Data from McBride *et al*. (2021).

Diet	Inclusion level of food waste ingredient	Global warming potential	Land use	Water consumption	Marine eutrophication
*Baseline*	*0%*	*100%*	*100%*	*100%*	*100%*
BSFL meal	5%	179%	86%	108%	98%
	10%	265%	73%	123%	100%
	15%	350%	66%	138%	102%
Food waste feed	5%	116%	90%	102%	94%
	10%	131%	79%	104%	88%
	15%	151%	68%	112%	85%
Bakery meal	5%	97%	96%	97%	96%
	10%	95%	92%	96%	93%
	15%	99%	92%	97%	90%

A more comprehensive LCA, covering 8,820 combinations in the European context, confirmed these findings (Javourez *et al*., 2025). It concluded that, unless used as a meat substitute, insects fed waste consistently showed higher environmental impacts than feeding waste directly to livestock. The higher impacts were attributed to conversion losses in the production chain, despite the higher protein content and digestibility of insect‐based feeds.

### (2) Other levers to improve the sustainability of insect farming

A comprehensive assessment of the potential of insect farming necessitates consideration of prospective improvements in production systems. Many of the studies analysed were conducted in small‐scale facilities, and technological advancements in production scalability could reduce environmental impacts (Halloran *et al*., 2016; Smetana *et al*., 2019,2021*b*; Wade & Hoelle, 2020; Quang Tran *et al*., 2022). Upscaling insect production could lead to more efficient resource use, and there is room for improvement, especially if insects are reared on non‐utilised side streams (Smetana *et al*., 2019; Bosch *et al*., 2019; Food Standards Agency, 2023). Small‐scale systems tend to be less sustainable than larger ones, although scale played a comparatively much smaller role compared to key factors such as heating and substrate type (Pahmeyer *et al*., 2022).

Ferronato *et al*. (2024) observed that overall environmental impact could be reduced by extending equipment lifespan by 15 years, and using products close to the treatment facility, as well as cutting energy use by 50% and stopping reliance on fossil energy. Switching to renewable energy is crucial to reduce GHG emissions (Maiolo *et al*., 2021), although ‘it is unlikely that on‐site renewables will be a solution for all insect producers’ (Smetana *et al*., 2019, p. 292). Furthermore, transitioning to renewable energy would benefit all livestock feed production methods, so insect farming does not have a particular advantage in this regard (Paris *et al*., 2022; Ryba, 2024).

The use of waste heat from other industries for insect facilities may reduce energy usage (Reyes‐Lúa *et al*., 2021). For instance, data centre excess heat has been used to provide high temperatures for mealworm farms (Vesterlund, Borisová & Emilsson, 2024). It showed promising results, although reductions in CO_2_ emissions and energy use were not quantified. Industrial symbiosis, where insect farms collaborate with other industries to utilise waste streams, also shows potential (Haq, Välisuo & Niemi, 2021). Of course, relying on industrial symbiosis will curtail the available options for the insect industry to locate and build new sites. Of the processing methods, in a recent study, ‘FOP (freezing–oven drying–hot pressing) showed the best environmental performance in terms of all selected impact categories except water use, while the BOS [blanching–oven drying–SFE (supercritical fluid extraction) with CO_2_] group had the highest environmental impacts in all categories’ (Cámara‐Ruiz *et al*., 2023, p. 1).

One potential solution to mitigate the environmental impact of heating is to use frass (insect waste) in biogas plants. This strategy proposes a circular energy model where frass is used to generate biogas, with the resultant exhaust heat redirected to maintain optimal temperatures in rearing facilities (Wedwitschka, Gallegos Ibanez & Jáquez, 2023; Abubakar *et al*., 2023). The economic viability and environmental contributions of this strategy are still under investigation.

Several innovations are being explored to reduce environmental impacts, such as pulsed electric fields, a pre‐treatment more energy‐efficient than blanching (Hajj, 2023). Some studies have explored the use of insect‐based milk, which has a lower environmental impact than bovine and soy milk, and a similar impact to almond and oat milk (Tello *et al*., 2021). It is likely, however, to face similar consumer acceptance issues as other insect food products. Other studies have explored laboratory‐grown insect meat, with similar benefits and challenges compared to normal insect meat substitutes, although energy consumption could be higher (Siddiqui *et al*., 2024*b*). Regarding the use of insects for human consumption, some studies suggest that integrating the cost of externalities into the price of food would make insects more competitive than some animal products (Xu & Milana, 2024).

One benefit to be explored further is the supplementation of fish or livestock diets with antimicrobial peptides (AMPs) naturally found in insects, which help protect against diseases and strengthen the immune system (Xia, Ge & Yao, 2021). Even small amounts of BSFL AMPs have been shown to reduce crayfish mortality (Zhang *et al*., 2024).

Some studies have explored whether genetic selection and modification in insects could represent a potential path toward improved sustainability (Athanassiou *et al*., 2024). Unlike conventional livestock, research on the genetics of BSFL is still in its early stages, with limited genomic resources available. Dossey *et al*. (2023) highlighted how gene editing in crickets could enhance growth rates or reduce mortality and disease. In terms of artificial selection, one study showed increased mass in BSFL, although there was no significant effect on GHG emissions, while noting possibilities for future improvements (Facchini *et al*., 2022). The success of genetically modified insects will depend on public acceptance; early genetically modified organism (GMO) companies, confident in the environmental benefits of genetic modification for crops and livestock, underestimated the impact of public opinion (Mohorčich & Reese, 2019; Food and Agriculture Organization, 2021; Ryba, 2024).

While various insect‐derived coproducts, such as lipids, chitin, pigments, nanochitin, chitosan, protein extracts, bioactive peptides and biofuel, are under investigation (Ravi *et al*., 2020; Moruzzo *et al*., 2021; Röthig *et al*., 2023; Hasnan *et al*., 2023), they fall outside our scope herein, which focuses on insects as food, feed, and fertilizer. These coproducts are still in the early stages of development, with limited environmental data available, warranting further research.

### (3) Future challenges

The question remains: can the insect farming sector realistically achieve the environmental benefits it claims? Answering this is complex, as predicting whether some practices will be economically viable in a new sector is fraught with uncertainty. This section discusses key challenges, informed by existing data gaps but limited by our unsuccessful attempts to obtain input from key industry players despite our efforts.

Studies suggest that realising the environmental potential of insect farming requires optimal conditions, including technological advancements, decarbonised energy sources, effective waste utilisation, and/or the replacement of meat in human diets (Smetana *et al*., 2023*a*; Javourez *et al*., 2025; Defra & Ricardo, 2024). As seen above, the sector could benefit from future technological advancements that may reduce production costs and improve scalability. It is possible that advancements in energy efficiency and waste processing could make these goals more achievable, although the timeline for such breakthroughs remains uncertain.

The greatest environmental benefits occur when insects replace meat, which has been described in one study as a ‘crucial prerequisite’ (Javourez *et al*., 2025). However, substituting meat is highly challenging, with low consumer acceptability and scepticism from investors and industry (Biteau *et al*., 2025). Future research should identify niches where insects could replace meat, if they exist, especially in areas where plant‐based alternatives are not accepted.

Regarding insect‐based feed, a recent LCA in the UK explored scenarios where insects might have lower climate impacts than soybean and fishmeal (Defra & Ricardo, 2024). However, this would require an ideal scenario involving decarbonised energy, utilising currently unauthorised waste in the UK and EU, or employing alternative technologies such as electric‐powered ovens for drying. While frass could play a future role in a circular economy, further research is needed to understand its real impact on plants and the environment. Since insects have a lesser impact on marine biodiversity, placing more weight on overfishing than on climate change or energy use could also make insects appear more beneficial. Innovations in alternative energy sources, drying technologies, and frass application could help overcome current barriers, although the feasibility of scaling up remains unclear.

To make the use of waste more widespread than it currently is, waste must meet several criteria: it must meet sanitary standards and legal requirements, be easy to collect, nutritionally stable, suitable for fast larval growth, and not usable as direct livestock feed. The right species (mainly BSFL) must also be used. While meeting all these constraints may be feasible in niche roles, scaling it up, especially in Western countries, remains highly challenging, particularly in an economically viable manner. Industry success may depend on whether waste‐sourcing logistics and regulatory frameworks become more flexible in the future. This remains uncertain but could be influenced by policy changes, such as the UK's household waste policy set to take effect in 2026, which aims to prevent organic waste from entering landfills and could shape how organic waste is processed and utilized in the future.

## X. ECONOMIC VIABILITY

Economic competitiveness is crucial, as many products and technologies considered more sustainable have failed to displace conventional ones because of their higher costs, such as green plastics or eco‐friendly insulation materials.

Consider the case of fishmeal, which is a major market targeted by insect producers. Unless insect meal becomes a more cost‐competitive option, it is unlikely to reduce the pressure on marine forage fish considerably. Currently, insect proteins tend to be considerably more expensive than fishmeal and this price disparity might persist into the future (de Jong & Nikolik, 2021).

The most extensive available model on the costs of production comes from Leipertz, Hogeveen & Saatkamp (2024), who state that, due to costs, “insects will likely not be part of mass farm animal feed in the near future.” (p. 23). Their default scenario for production in the Netherlands gives a production price for defatted larvae meal of 5,116 EUR per ton of dry matter (approximately 5,500 USD). This price is significantly higher than the competitive price point needed to rival conventional protein sources like fishmeal, which is around 1,296 EUR (1,400 USD).

Leipertz *et al*. (2024) also explore future scenarios, concluding that simply improving production processes and parameters will not suffice to make insect farming cost‐competitive with fishmeal. They suggest that profitability might be achieved by selling frass at a high price or by acquiring feeding substrates at very low costs, such as waste. However, these conditions may be unrealistic for mass production (Leipertz *et al*., 2024). Other studies also list significant barriers to economic competitiveness (Caparros Megido *et al*., 2024; Biteau *et al*., 2024*a*). A good nutritional profile is insufficient to make insects a large part of sustainable aquaculture (Panteli *et al*., 2025).

A series of interviews with insect‐rearing experts indicate that “currently the insect industry is not able to offer many economic, environmental or social values”, and that barriers such as food acceptance or regulation need to be overcome before creating positive impact (Bijvoet, 2022, p. 1). Industry analysts from Rabobank, who previously identified fish feed as the largest market for edible insects, now report that ‘the fish feed industry uses virtually no insect protein’ due to high costs and inconsistent supply (van der Velden, van Houting & Blankestijn, 2025). The FAIRR initiative also reports that of the seven top salmon companies they engaged with, four saw insect meal as “unfavourable” (Boissat, Boucher & Finkelstein, 2025). Given these challenges, the International Platform of Insects for Food and Feed (IPIFF) recently stated that it ‘does not claim to replace soy or fish meals used in conventional farming but rather to offer a complementary product for certain farmers, at a premium price justified by its high content of proteins, vitamins, and minerals’ (Playoust‐Braure, 2024, paragraph 7). If insect meal is just a complement, this puts into question the claims that it could significantly reduce pressures on marine biodiversity and deforestation.

Economic challenges are also present in other markets. Although insect‐based pet food is the most profitable market segment, this is because it targets premium consumers, such as those seeking hypoallergenic ingredients, and who are willing to pay higher prices (de Jong & Nikolik, 2021). Insect frass currently is 4–10 times more expensive than other organic fertilisers, such as compost and manure (Kragt *et al*., 2023). Finally, as meat substitutes, insect‐based products are more expensive than most alternatives (Malila *et al*., 2024), facing significant price penalties (Michel & Begho, 2023).

## XI. CONCLUSIONS

(1) We critically examined the scientific literature on the environmental impacts of insect farming. The sustainability of insect production is influenced by several factors, including the type of substrates used, production location, scale, insect species, and the specific end‐use products they replace.
(2) While insect farming appears to hold potential for contributing positively to a circular economy, particularly due to its ability to process waste and substitute meat, in practice, the industry's current trajectory in Western countries does not align with these goals, primarily due to substantial economic and technical challenges. For example, the limited use of waste substrates undermines the environmental case for insect farming.
(3) In the food sector, only about 10% of insect‐based products replace meat, with the majority substituting for plant‐based items or being incorporated into products like cookies, snacks, and pasta. While studies are lacking, including insects in these products likely increases their environmental impacts. While insects can be more sustainable than conventional livestock, current data suggest they do not offer significant advantages over plant‐based meat substitutes, which tend to have a lower environmental footprint and higher consumer acceptance.
(4) For insect‐based pet food, which accounts for half of the insect farming market, the sparse data indicate a significantly higher carbon footprint compared to conventional products.
(5) There are concerns about the potential risks to local biodiversity from escaped insects, particularly regarding the spread of pathogens, parasites, and modified genes.
(6) Most LCAs indicate that insect farming has a higher climate impact than soybean meal or fishmeal when food waste is not used as a substrate, and in some studies even when waste is used. While insect‐based feeds could potentially reduce forage fish depletion or, in some cases, land use, their high production costs and scalability issues limit our ability to reduce reliance on conventional feeds in the near term. Alternative solutions, such as sustainably sourced soy or innovative fish feed formulations, appear to be more promising for reducing the environmental footprint of the food system.
(7) The use of insect frass as a fertiliser offers potential benefits for plant growth and resilience. However, due to the large variability in frass composition, a lack of field studies, and conflicting results regarding its impact on soils, the usefulness and safety of frass at a large scale remain uncertain. Further research is needed to assess its environmental impact, particularly when treated, and to determine whether high GHG emissions from soils persist.
(8) Despite these challenges, some improvements are possible. Insect‐based products could be environmentally beneficial where production systems are highly efficient (Smetana *et al*., 2023*a*). The use of waste may also be an important benefit. Technological advancements, such as heat recovery from nearby industrial processes, could help reduce environmental impacts. Depending on economic viability, insects could play a role in waste management on small scales, especially with local substrates. The industry remains in its infancy, and while the versatility of insects presents potential, predicting which applications will be economically viable and sustainable at scale remains complex.
(9) There are significant knowledge gaps in the literature. Future research that addresses these gaps, specifically comprehensive evaluations of insects farmed under industrial‐scale conditions for realistic end‐use products, would provide a clearer understanding of the prospects for insect farming.
(10) Future studies should explore the environmental impacts of insect‐based foods that do not replace meat, such as snacks, pasta, and bars, and compare insect‐based meat substitutes with alternative proteins. In addition, the environmental impact of insect pet foods should be assessed. More data are required on the risks to local biodiversity and the potential ecological impact of frass following heat treatment. Additional LCAs are needed, with substrates representative of industry conditions.
(11) While specific applications of insect farming show potential, the available evidence suggests that, in most cases, its impact on the sustainability of the food system is less positive than initially promised.

## AUTHOR CONTRIBUTIONS

Conceptualization: C. B., T. B.‐C, D. C., M. S. J.; Investigation: C. B., T. B.‐C.; Writing – Original Draft: C. B.; Writing – Review & Editing: C. B., T. B.‐C., D. C., K. L., R. R., M. S. J.; Visualization: D. C., R. R.; Supervision: D. C.

## Data Availability

Data sharing is not applicable to this article as no new data were created or analysed in this study.
